# Regioselective Fluorination of 7-Oxo-1,2,4-benzotriazines Using Selectfluor

**DOI:** 10.3390/molecules24020282

**Published:** 2019-01-14

**Authors:** Styliana I. Mirallai, Panayiotis A. Koutentis, Fawaz Aldabbagh

**Affiliations:** 1 School of Chemistry, National University of Ireland Galway, University Road, H91 TK33 Galway, Ireland; styliana.mirallai@nuigalway.ie; 2 Department of Chemistry, University of Cyprus, P.O. Box 20537, Nicosia 1678, Cyprus; koutenti@ucy.ac.cy

**Keywords:** iminoquinone, enamine, fluorine, heterocyclic compound, microwave

## Abstract

7-Oxo-1,2,4-benzotriazines (benzo[1,2,4]triazin-7-ones) are reversible thioredoxin reductase inhibitors that exhibit very strong correlations to pleurotin. In this article, we provide the first synthesis of fluorinated derivatives. Fluorination using Selectfluor of benzo[1,2,4]triazin-7-ones occurs regioselectively and in high yield at the enamine-activated position. This electron N-lone pair activation overrides the activation/deactivation effects of some other substituents. The reaction time was significantly reduced with the use of microwave irradiation at 120 °C and 7 bar. The cytotoxicity and cyclic voltammetry measurements for 8-fluoro-1,3-diphenylbenzo[*e*][1,2,4]triazin-7(1*H*)-one (**2**) are presented and compared with its synthetic precursor, 1,3-diphenylbenzo[*e*][1,2,4]triazin-7(1*H*)-one (**1a**).

## 1. Introduction

**Figure 1:**
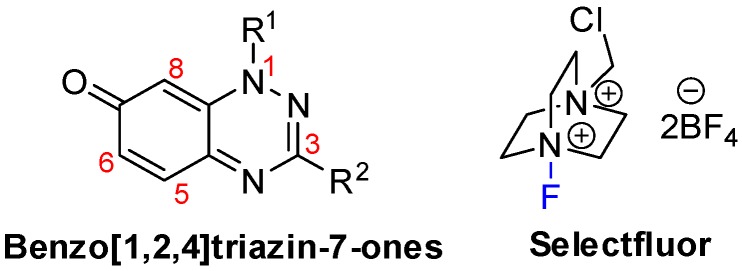
Key chemical structures.

The remarkable expansion in the use of fluorinated chemicals has attracted the attention of organic, agricultural, medicinal, and materials scientists. Uniquely, the incorporation of fluorine atoms into organic molecules introduces polar hydrophobicity. This has been significantly utilized in medicinal chemistry as a means to increase efficacy [1,2], with some 30% of blockbuster drugs containing fluorine atoms [3]. Selectfluor is perhaps the most versatile, stable, cheap, and effective commercial electrophilic fluorinating reagent [4], and in this article, we demonstrate its use in the selective fluorination of some 7-oxo-1,2,4-benzotriazines (benzo[1,2,4]triazin-7-ones) (Figure 1). Selectfluor is also a strong oxidant, and mediator or catalyst of several “fluorine-free” transformations [5]. Selectfluor is known to directly substitute fluorine into activated positions on anilines, benzamides, and phenols [6,7]; however, to date, examples of fluorinated quinones are few and low-yielding (<20%) [8,9]. Typically, in such cases, the fluorine was introduced via a halogen exchange with nucleophilic fluoride reagents, such as KF [10,11,12]. Interestingly, 2-hydroxymethylindole can undergo simultaneous electrophilic aromatic substitution with fluorine at the activated enamine C-3 position with oxidation of the alcohol to the aldehyde by using Selectfluor [13].

Benzo[1,2,4]triazin-7-ones are reversible thioredoxin reductase inhibitors with significant anti-cancer activities, which strongly correlate to pleurotin [14]. Derivatives also exhibit anti-Alzheimer’s disease activity [15], while the cytotoxicity is far greater than that of the derived Blatter-type (benzotriazin-4-yl) radicals [16]. Herein, we present the first fluorination of several benzo[1,2,4]triazin-7-one derivatives (that were available to us). Additionally, the cytotoxicity and cyclic voltammetry of 8-fluoro-1,3-diphenylbenzo[*e*][1,2,4]triazin-7(1*H*)-one (**2**) is compared to that of the non-fluorinated scaffold **1a**.

## 2. Results and Discussion

### 2.1. Fluorinations

#### 2.1.1. Optimizing the Fluorination and Confirming Selectivity

**Scheme 1:**
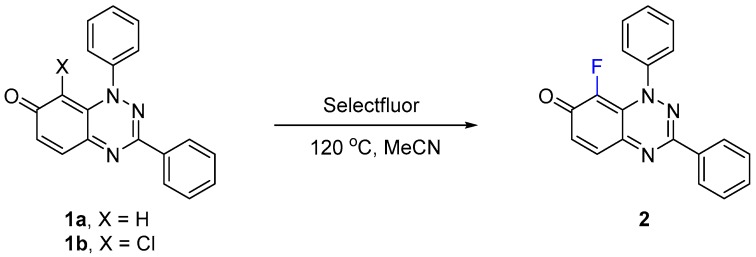
Synthesis of 8-fluoro-1,3-diphenylbenzo[*e*][1,2,4]triazin-7(1*H*)-one (**2**).

Selectfluor was found to fluorinate at the 8-position of the parent 1,3-diphenylbenzo[*e*][1,2,4]triazin-7(1*H*)-one (**1a**), presumably due to enamine conjugation with the N-1 atom (Scheme 1). Initially, low yields of 8-fluoro derivative **2** were obtained, when using less than 2 equivalents of Selectfluor at room temperature and at reflux in acetonitrile, with recovery of **1a** (Table 1). The complete conversion of **1a** to **2** was observed by TLC after 1 h when increasing the reaction temperature to 120 °C, which was facilitated by using a sealed Ace pressure tube with **2** isolated in 94% yield after column chromatography. The reaction time was reduced to 20 min when performing the transformation in a microwave reactor (150 W, 7 bar), with **2** isolated at 97% yield. Applying the latter optimized conditions on 8-chloro-1,3-diphenylbenzo[*e*][1,2,4]triazin-7(1*H*)-one (**1b**), a halogen exchange proceeds efficiently yielding **2** in 96% yield, although after a longer reaction time of 1 h, as monitored by TLC. Jiang et al. [13] reported a similar aromatic substitution of bromine by fluorine at the C-3 position of indole, using Selectfluor. In these cases, the defluorinated Selectfluor by-product (1-chloromethyl-1,4-diazobicyclo[2.2.2]octane) may assist with the elimination of the halogen atom.

**Figure 2:**
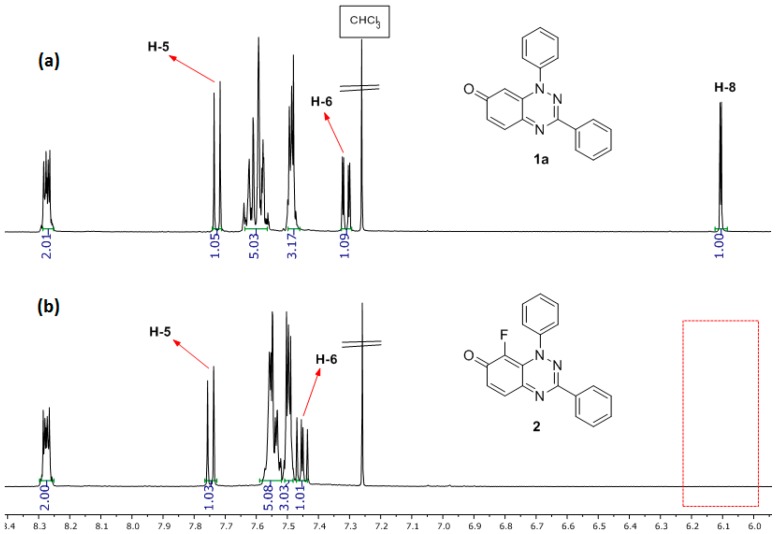
^1^H-NMR spectrum: (**a**) of 1,3-diphenylbenzo[*e*][1,2,4]triazin-7(1*H*)-one (**1a**) and (**b**) of 8-fluoro-1,3-diphenylbenzo[*e*][1,2,4]triazin-7(1*H*)-one (**2**).

The position of the fluorination on **2** is discernible by comparing its ^1^H-NMR spectrum with that of substrate **1a** (Figure 2) with the disappearance of the H-8 signal of **1a** at 6.10 ppm being clearly visible, and ^1^H-^19^F (*meta*) coupling for H-6 of *J* = 7.2 Hz. The ^13^C-NMR spectrum of **2** gives the expected ^13^C-^19^F couplings, including ^1^*J*_CF_ of 247.5 Hz for C-8, and ^2^*J*_CF_ of 15.7 Hz for C=O.

#### 2.1.2. Fluorination of C-6-Substituted Benzo[1,2,4]triazin-7-ones

**Scheme 2:**
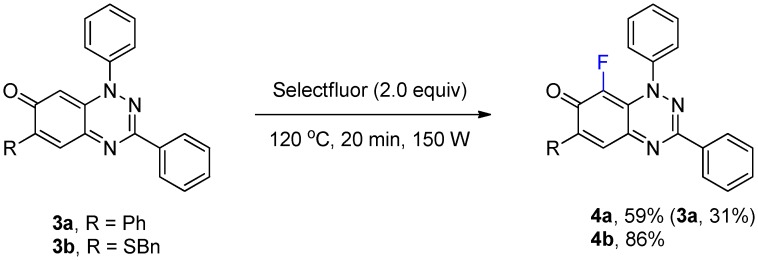
Synthesis of 8-fluoro-1,3,6-triphenylbenzo[*e*][1,2,4]triazin-7(1*H*)-one (**4a**) and 6-(benzylthio)-8-fluoro-1,3-diphenylbenzo[*e*][1,2,4]triazin-7(1*H*)-one (**4b**).

The optimized conditions in Table 1 of treatment with Selectfluor (2 equiv, MW at 120 °C, 20 min) were applied to benzo[1,2,4]triazin-7-ones **3a** and **3b** containing phenyl and benzylthio substituents at C-6 (Scheme 2). C-8 Fluorinated derivatives **4a** and **4b** were isolated in 59% and 86% yield, respectively, with **4a** accompanied by significant recovery of **3a** (31%). Interestingly, fluorination at C-8 remained the only path for substitution despite activation at C-5 by the benzylthio substituent of **3b**.

#### 2.1.3. Fluorination of 1,2,5-Thiadiazolo-Fused Benzotriazinones

**Scheme 3:**
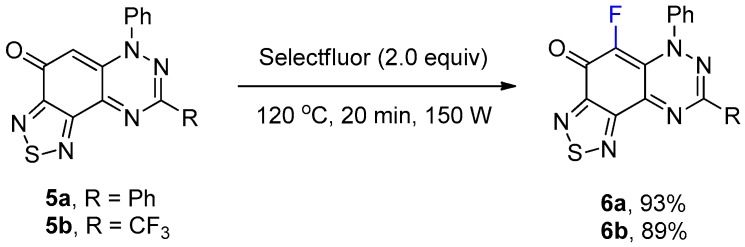
Synthesis of 5-fluoro-6,8-diphenyl[1,2,5]thiadiazolo[3′,4′:5,6]benzo[1,2-*e*][1,2,4]triazin-4(6*H*)-one (**6a**) and 5-fluoro-6-phenyl-8-(trifluoromethyl)[1,2,5]thiadiazolo[3′,4′:5,6]benzo[1,2-*e*][1,2,4]triazin-4(6*H*)-one (**6b**).

The preparation of 1,2,5-thiadiazolo-fused benzotriazinones **5a** and **5b** has been recently reported by using the reaction of S_4_N_4_ with **1a** and the 3-trifluoromethyl analogue [17]. The reaction of thiadiazoles **5a** and **5b** with Selectfluor using the optimized conditions in Table 1 gave the fluorinated adducts **6a** (93%) and **6b** (89%) in excellent yields, where the reaction occurred at the only available CH of the benzotriazinone scaffold (Scheme 3). The regioselective formation of **6b** in high yield demonstrated that the electrophilic fluorination remained facile, despite the strongly deactivating inductive effect of the CF_3_ substituent of **5b**.

The NMR data for fluorinated adducts revealed a “through space” coupling with the *ortho*-CH on the N-1-Ph, supported by distance measurements using Spartan (Figure S16).

### 2.2. Cytotoxicity against MCF-7 using the MTT Assay

Having the fluorinated benzotriazin-7-one **2**, we investigated the effect of the fluorine substituent on cytotoxicity. The breast cancer cell line MCF-7, and the MTT [3-(4,5-dimethylthiazol-2-yl)-2,5-diphenyltetrazolium bromide] assay were employed by using the conditions previously described on the parent compound **1a** [14]. The fluorinated derivative **2** proved to be approximately five times more cytotoxic than **1a** towards the MCF-7 cell line (Table 2).

### 2.3. Cyclic Voltammetry

**Figure 3:**
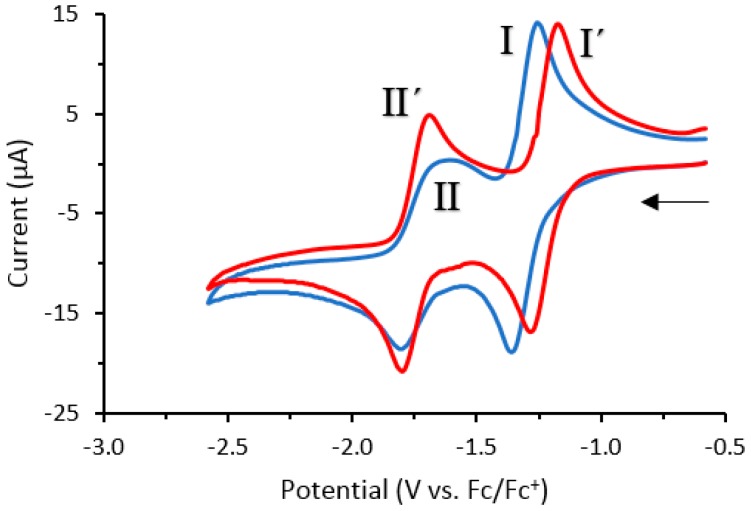
Cyclic voltammograms of **1a** (blue line) and **2** (red line) recorded in CH_2_Cl_2_ (0.1 M *n*-Bu_4_NPF_6_) at a glassy carbon electrode (scan rate: 0.1 V·s^−1^). Arrow indicates the direction of the scan.

Cyclic voltammetry studies were carried out on compounds **1a** and **2** (Figure 3). Redox response experiments showed that both **1a** and **2** undergo two characteristic quasi-reversible one-electron redox processes corresponding to the 0/−1 redox transition (I and I′) and the −1/−2 redox transition (II and II′). The fluorinated derivative **2** produced a similar redox response to **1a**, with surprisingly similar formal potentials (*E*^0^′) (Table 3), despite the presence of the electronegative fluorine at the C-8 position. This indicates that factors other than bioreduction may account for the differences in cytotoxicity between **1a** and **2** against the MCF-7 cell line.

## 3. Experimental Section

### 3.1. General Materials and Methods

1,3-Diphenylbenzo[*e*][1,2,4]triazin-7(1*H*)-one (**1a**) [18,19] and 1,3,6-triphenylbenzo[*e*][1,2,4]-triazin-7(1*H*)-one (**3a**) [18] were prepared according to literature procedures. 8-Chloro-1,3-diphenyl-benzo[*e*][1,2,4]triazin-7(1*H*)-one (**1b**) [18], 6-(benzylthio)-1,3-diphenylbenzo[*e*][1,2,4]triazin-7(1*H*)-one (**3b**) [18], 6,8-diphenyl[1,2,5]thiadiazolo[3′,4′:5,6]benzo[1,2-*e*][1,2,4]triazin-4(6*H*)-one (**5a**) [17] and 6-phenyl-8-(trifluoromethyl)-[1,2,5]thiadiazolo[3′,4′:5,6]benzo[1,2-*e*][1,2,4]triazin-4(6*H*)-one (**5b**) [17] were provided by Koutentis Research Laboratory (University of Cyprus), and used as received. All other solvents and reagents were used as received from Sigma-Aldrich (Gillingham, Dorset, SP8 4XT, UK). Acetonitrile (MeCN, Sigma-Aldrich, ≥99.9%) was freshly distilled over 3 Å molecular sieves and then over CaH_2_ (Sigma-Aldrich, 95%). Thin-layer chromatography (TLC) was performed on Merck TLC Silica gel 60 F_254_ plates using a UV lamp for visualization. The technique of dry flash chromatography [20] was used throughout for all non-TLC-scale chromatographic separations using silica gel 60 (<0.063 mm). Microwave irradiation was conducted in a CEM Discover SP Microwave Reactor using 150 watts of microwave power. The pressure was controlled by a load cell connected to the vessel via the cap on top of the sealed pressure vessel. The temperature of the content of the vessel was monitored by an infrared temperature control system, which uses a non-contact, infrared sensor mounted under the vessel. All reactions were performed in Pyrex pressure vessels (capacity 10 mL) sealed with silicone caps. All reaction mixtures were stirred with a Teflon-coated, magnetic stirring bar in the vessel. A ramp temperature of 2 min was set for each experiment. Ultraviolet spectra were obtained on a Varian (Cary 100) UV-Vis spectrometer, where inf = inflection. Infrared spectra were recorded using a PerkinElmer Spec 1 with attenuated total reflection (ATR) attached. NMR spectra were recorded using Varian 500 MHz, and an Agilent DD2 600 MHz instrument was used to be obtain the ^13^C-NMR spectrum of compound **6b**. The chemical shifts were recorded in ppm relative to SiMe_4_. ^13^C-NMR data were collected at 125 MHz and 150 MHz for compound **6b** with complete proton decoupling. NMR assignments were supported by distortionless enhancement by polarization transfer (DEPT). ^19^F-NMR spectra were obtained at 470 MHz. Deuterated solvents were used for the homonuclear lock, and the signals were referenced to the deuterated solvent peaks. High resolution mass spectra (HRMS) was carried out using an ESI time-of light mass spectrometer (TOFMS) in positive or negative mode, using a Waters LCT Mass Spectrometry instrument. The precision of all accurate mass measurements was better than 5 ppm. Melting points were determined by using differential scanning calorimetry (DSC), which was performed on a Mettler Toledo Simultaneous Thermal Analyzer using standard aluminium pans.

### 3.2. Synthetic Procedures and Characterization


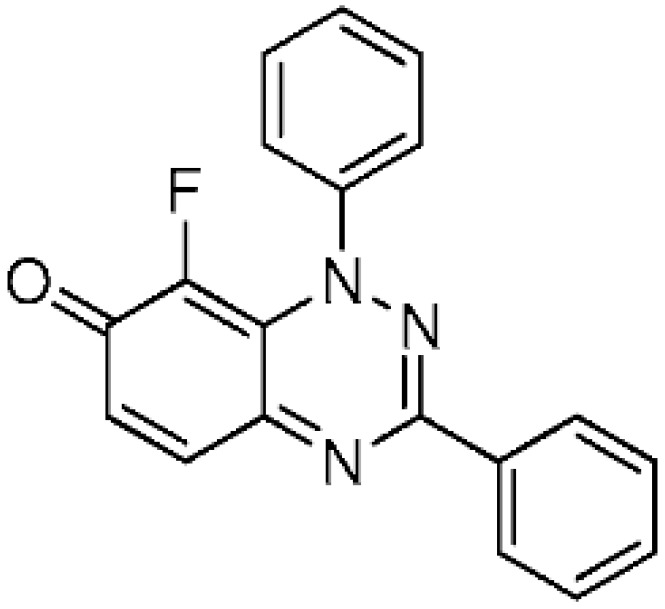


Method A: Selectfluor (238.2 mg, 0.8 mmol) was added to the solution of 1,3-diphenylbenzo[*e*][1,2,4]triazin-7(1*H*)-one (**1a**) (120 mg, 0.4 mmol) in dry MeCN (2 mL) in an Ace pressure tube (15 mL). The reaction mixture was immersed in a preheated oil bath at ca. 120 °C and left to stir for 1 h, under monitoring by TLC. The reaction mixture was cooled to ca. 20 °C, diluted with EtOAc (30 mL), and washed with brine (3 × 30 mL). The organic layer was separated, dried over anhydrous MgSO_4_, filtered, and evaporated to dryness. The residue was purified by column chromatography using EtOAc and petroleum ether to give 8-fluoro-1,3-diphenylbenzo[*e*][1,2,4]-triazin-7(1*H*)-one (2) (119.3 mg, 94%) as blue fine needles; m.p. (DSC) onset 202.3 °C, peak max 205.2 °C (from cyclohexane/CH_2_Cl_2_, 90:10); *R*_f_ 0.42 (EtOAc/Petroleum ether, 60:40); *λ*_max_(CH_2_Cl_2_)/nm 300 (log *ε* 5.12), 350 inf (4.69), 360 inf (4.65), 575 (4.29), 615 inf (4.25), 680 inf (3.84); *ν*_max_ (neat, cm^−1^) 3057, 1608 (C=O), 1545, 1491, 1436, 1377, 1344, 1211, 1160, 955; ^1^H-NMR (500 MHz, CDCl_3_) *δ*_H_ 7.45 (1H, dd, *J* = 7.2 Hz, *J =* 9.8 Hz, H-6), 7.48–7.51 (3H, m), 7.52–7.57 (5H, m), 7.75 (1H, d, *J* = 9.8 Hz, H-5), 8.26–8.29 (2H, m); ^13^C-NMR (125 MHz, CDCl_3_) *δ*_C_ 121.2 (d, *J* = 3.8 Hz, C), 124.8 (d, *J* = 4.2 Hz, CH), 126.8, 128.9 (×2), 129.6, 130.7, 130.9 (all CH), 133.4 (C), 136.1 (d, ^1^*J*_CF_ = 247.5 Hz, CF), 141.3 (CH), 143.6 (d, *J* = 2.7 Hz, C), 150.9, 153.9 (both C), 172.7 (d, *J* = 15.7 Hz, C=O); ^19^F-NMR (470 MHz, CDCl_3_) *δ*_F_ −145.8 (1F, s); HRMS (ESI) *m*/*z* [M + H]^+^, C_19_H_13_FN_3_O calcd. 318.1043, observed 318.1054.

Method B (General Procedure): Selectfluor (70.8 mg, 0.2 mmol) was added to the solution of the benzo[*e*][1,2,4]triazin-7(1*H*)-one **1a**–**1b**, **3a**–**3b**, **5a**–**5b** (0.1 mmol) in dry MeCN (0.5 mL). The reaction mixture was stirred under microwave irradiation (150 W, ca. 120 °C, 7 bar) for 20 min (1 h in the case of **1b**). EtOAc (10 mL) was added and the mixture was extracted with brine (3 × 10 mL). The organic layer was separated, dried over anhydrous MgSO_4_, filtered, and evaporated to dryness. The residue was dissolved in CH_2_Cl_2_, poured onto a short pad of silica, and washed with EtOAc and petroleum ether to give the desired products **2**, **4a**–**4b**, and **6a**–**6b**.


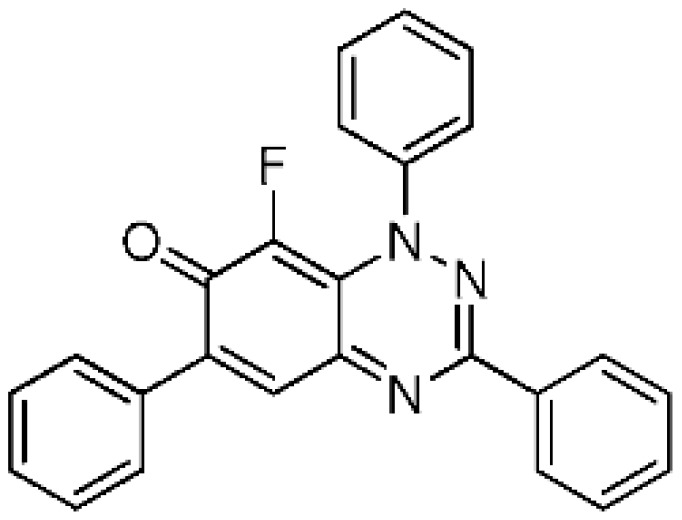


8-Fluoro-1,3,6-triphenylbenzo[*e*][1,2,4]triazin-7(1*H*)-one (**4a**) (23.4 mg, 59%) as blue fine needles; m.p. (DSC) onset 252.0 °C, peak max 252.8 °C (from cyclohexane/CH_2_Cl_2_, 90:10); *R*_f_ 0.50 (EtOAc/petroleum ether, 30:70); *λ*_max_(CH_2_Cl_2_)/nm 260 (log *ε* 5.03), 330 (4.98), 410 inf (4.13), 595 (4.06), 670 inf (3.76); *ν*_max_ (neat, cm^−1^) 3068, 1605 (C=O), 1542, 1491, 1424, 1312, 1281, 1210, 1160, 1108, 962; ^1^H-NMR (500 MHz, CDCl_3_) *δ*_H_ 7.47–7.62 (11H, m), 7.79–7.82 (2H, m), 7.88 (1H, s, H-5), 8.29–8.31 (2H, m); ^13^C-NMR (125 MHz, CDCl_3_) *δ*_C_ 120.5 (d, *J* = 4.2 Hz, C), 125.1 (d, *J* = 4.3 Hz, 5-CH), 126.9, 127.8, 128.6, 129.0 (×2), 129.6, 129.7, 130.2, 130.8 (all CH), 134.0 (C), 134.9 (d, *J* = 2.3 Hz, C), 136.3 (d, ^1^*J*_CF_ = 244.6 Hz, CF), 143.8 (C), 150.6 (d, *J* = 3.2 Hz, C), 151.2, 153.4 (both C), 171.9 (d, *J* = 15.7 Hz, C=O); ^19^F-NMR (470 MHz, CDCl_3_) *δ*_F_ −145.0 (1F, s); HRMS (ESI) *m*/*z* [M + H]^+^, C_25_H_17_FN_3_O calcd. 394.1356, observed 394.1349. Further elution with EtOAc and petroleum ether (30:70), gave the recovered starting material **3a** (11.6 mg, 31%).


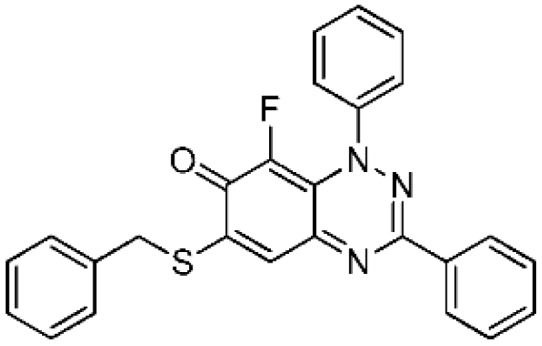


6-(Benzylthio)-8-fluoro-1,3-diphenylbenzo[*e*][1,2,4]triazin-7(1*H*)-one (**4b**) (38.0 mg, 86%) as olive green fine needles; m.p. (DSC) onset 217.5 °C, peak max 219.2 °C (from cyclohexane/CH_2_Cl_2_, 90:10); *R*_f_ 0.43 (EtOAc/petroleum ether, 30:70); *λ*_max_(CH_2_Cl_2_)/nm 260 (log *ε* 4.95), 325 (4.82), 410 inf (4.27), 425 (4.31), 575 (3.95), 635 inf (3.72); *ν*_max_ (neat, cm^−1^) 3061 (Ar CH), 1610 (C=O), 1574, 1523, 1491, 1430, 1311, 1281, 1214, 1112, 1072, 974; ^1^H-NMR (500 MHz, CDCl_3_) *δ*_H_ 4.29 (2H, s, CH_2_), 7.33–7.40 (3H, m), 7.46–7.52 (5H, m), 7.53–7.60 (6H, m), 8.26–8.32 (2H, m); ^13^C-NMR (125 MHz, CDCl_3_) *δ*_C_ 36.2 (CH_2_), 118.7 (CH), 120.8 (d, *J* = 3.1 Hz, C), 125.1 (d, *J* = 4.2 Hz, 5-CH), 127.0, 128.1, 128.9, 129.0, 129.1 (×2), 129.7, 130.8 (all CH), 133.6, 134.1 (both C), 134.6 (d, ^1^*J*_CF_ = 245.0 Hz, CF), 143.9 (d, *J* = 2.9 Hz, C), 150.5, 151.6 (both C), 158.4 (d, *J* = 4.6 Hz, C), 168.7 (d, *J* = 16.4 Hz, C=O); ^19^F-NMR (470 MHz, CDCl_3_) *δ*_F_ −147.5 (1F, s); HRMS (ESI) *m*/*z* [M + H]^+^, C_26_H_19_FN_3_OS calcd. 440.1233, observed 440.1220.


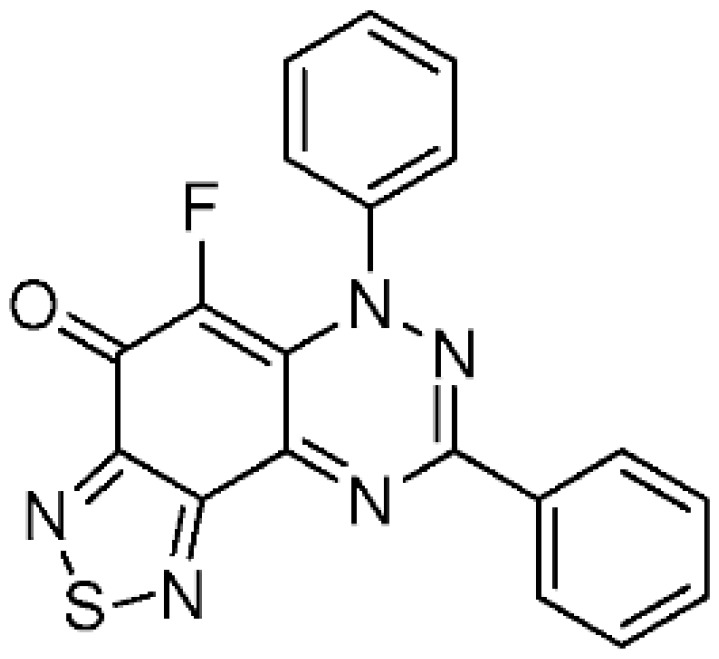


5-Fluoro-6,8-diphenyl[1,2,5]thiadiazolo[3′,4′:5,6]benzo[1,2-*e*][1,2,4]triazin-4(6*H*)-one (**6a**) (35.1 mg, 93%) as brown fine needles; m.p. (DSC) onset 315.7 °C, peak max 316.7 °C (from cyclohexane/CH_2_Cl_2_, 90:10); *R*_f_ 0.38 (EtOAc/petroleum ether, 40:60); *λ*_max_(CH_2_Cl_2_)/nm 275 (log *ε* 4.94), 310 (5.09), 430 (4.74), 515 inf (3.95), 555 (4.04), 600 inf (3.97), 660 inf (3.54); *ν*_max_ (neat, cm^−1^) 3068 (Ar CH), 1624 (C=O), 1573, 1534, 1464, 1434, 1345, 1238, 1198, 1161, 1061, 922; ^1^H-NMR (500 MHz, CDCl_3_) *δ*_H_ 7.50–7.58 (8H, m), 8.36–8.38 (2H, m); ^13^C-NMR (125 MHz, CDCl_3_) *δ*_C_ 124.7 (d, *J* = 4.6 Hz, CH), 125.0 (d, *J* = 5.3 Hz, C), 127.2, 129.1, 129.2, 129.9, 131.6 (all CH), 132.8 (C), 138.1 (d, ^1^*J*_CF_ = 249.3 Hz, CF), 143.6 (d, *J* = 2.9 Hz, C), 147.8, 151.2, 151.6 (all C), 156.6 (d, *J* = 7.8 Hz, C), 165.1 (d, *J* = 18.4 Hz, C=O); ^19^F-NMR (470 MHz, CDCl_3_) *δ*_F_ −145.5 (1F, s); HRMS (ESI) *m*/*z* [M + H]^+^, C_19_H_11_FN_5_OS calcd. 376.0668, observed 376.0654.


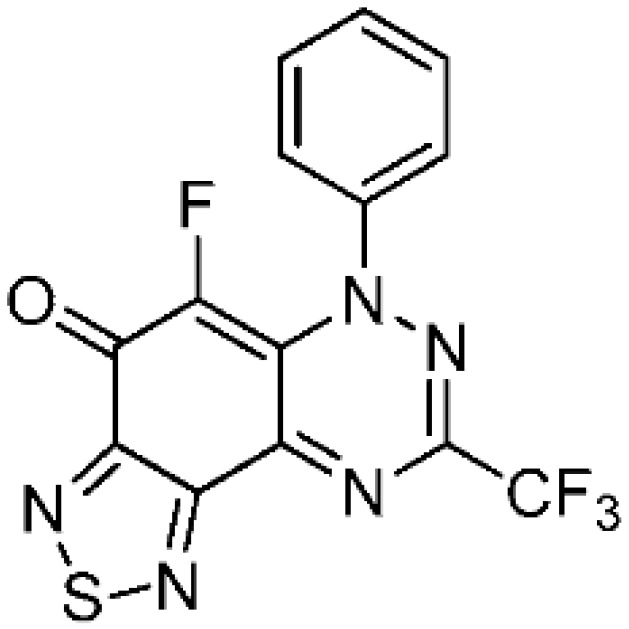


5-Fluoro-6-phenyl-8-(trifluoromethyl)[1,2,5]thiadiazolo[3′,4′:5,6]benzo[1,2-*e*][1,2,4]triazin-4(6*H*)-one (**6b**) (32.5 mg, 89%) as purple fine needles; m.p. (DSC) onset 279.0 °C, peak max 281.5 °C (from cyclohexane/CH_2_Cl_2_, 90:10); *R*_f_ 0.59 (EtOAc/petroleum ether, 40:60); *λ*_max_(CH_2_Cl_2_)/nm 260 (log *ε* 4.93), 300 (4.96), 315 inf (4.90), 325 inf (4.76), 390 inf (4.52), 405 (4.53), 500 inf (4.02), 540 (4.09), 590 inf (3.97), 645 inf (3.57); *ν*_max_ (neat, cm^−1^) 3072 (Ar CH), 1652 (C=O), 1587, 1567, 1487, 1414, 1387, 1294, 1204, 1131, 1068, 928; ^1^H-NMR (500 MHz, CDCl_3_) *δ*_H_ 7.49–7.53 (2H, m), 7.54–7.59 (3H, m); ^13^C-NMR (150 MHz, CDCl_3_) *δ*_C_ 119.0 (q, ^1^*J*_CF_ = 272.4 Hz, F_3_C), 124.1 (d, *J* = 5.8 Hz, C), 124.4 (d, *J* = 4.5 Hz, CH), 129.5, 130.4 (both CH), 138.4 (d, ^1^*J*_CF_ = 255.7 Hz, C-8), 142.6 (d, *J* = 2.7 Hz, C), 142.9 (q, *J* = 39.4 Hz, F_3_C*C*), 149.3, 150.8 (both C), 156.0 (d, *J* = 7.5 Hz, C), 165.9 (d, *J* = 19.5 Hz, C=O); ^19^F-NMR (470 MHz, CDCl_3_) *δ*_F_ −70.1 (3F, s, CF_3_), −141.3 (1F, s); HRMS (ESI) *m*/*z* [M + H]^+^, C_14_H_6_F_4_N_5_OS calcd. 368.0229, observed 368.0230.

### 3.3. Cell Culture and Cytotoxicity Evaluation

#### 3.3.1. Materials and Cell Lines

MCF-7 were cultured in Dulbecco’s modified Eagle’s medium (DMEM) containing high glucose (4.5 g/mL) and supplemented with 1% penicillin-streptomycin and 10% heat-inactivated foetal bovine serum (FBS). Cells grew as adherent cultures. Cell culture reagents were obtained from Sigma-Aldrich. Disposable sterile plasticware was obtained from Sarstedt (Numbrecht, Germany).

#### 3.3.2. Cytotoxicity Measurements Using the MTT Assay

The MTT colorimetric assay was used to determine cell viability. MCF-7 cells were added to 96-well plates at a cell density of 1000 cells per well (200 µL per well) and allowed to adhere over 24 h. Compound solutions in DMSO were added after 24 h (1% *v/v* final concentration in the well). The control cells were exposed to the same concentration of the vehicle control alone (DMSO). All cells were incubated at 37 °C and 5% CO_2_ (humidified atmosphere) for 72 h. MTT (20 µL, 5 mg/mL solution) was added after 72 h and the cells were incubated for a further 3 h. The supernatant was then removed by using a multi-transfer pipette, and DMSO (100 µL) was added to dissolve the MTT formazan crystals. The absorbance was determined by using a plate reader at 550 nm with a reference at 690 nm. Cell viability is expressed as a percentage of the vehicle-only treated control (DMSO). Dose-response curves were analysed by non-linear regression analysis, and IC_50_ values were determined by using GraphPad Prism software, v 8.0 (GraphPad Inc., San Diego, CA, USA). The in vitro activity of the drugs towards all cell lines is expressed as IC_50_ (i.e., the concentration required for the reduction of the mean cell viability to 50%).

### 3.4. Electrochemistry

Cyclic voltammograms were recorded using a PalmSens3+ potentiostat. The concentrations of all studied compounds were 0.001 mol·L^−1^ in dry (over CaH_2_) HPLC grade CH_2_Cl_2_ (5.0 mL) containing *n*-Bu_4_NPF_6_ (0.1 M) as a supporting electrolyte. A three-electrode electrochemical cell was employed with glassy carbon, Pt wire, and Ag/AgCl (1 M NaCl) as the working, counter, and reference electrodes, respectively. The ferrocene/ferrocenium (Fc/Fc^+^) couple was used as an internal reference, and all redox couples are referenced against it (*E*_Fc/Fc+_ 0.0 V). The scan rate was 0.1 V·s^−1^ and the temperature was 20 °C.

## 4. Conclusions

Selectfluor regioselectively fluorinates benzo[1,2,4]triazin-7-ones and derivatives in high yield. One of the fluorinated iminoquinones is shown to be cytotoxic, offering the potential for further investigation of fluorinated adducts as bioreductive antibiotics and anti-cancer agents.

## Figures and Tables

**Table 1:** Optimization of reaction conditions ^a^.

Entry	Iminoquinone	Selectfluor (equiv)	Time (h)	Isolated Yield 2 (%)
1	**1a**	1.2	24	32 ^b^
2	**1a**	1.5	24	55 ^c^
3	**1a**	2	1	94 ^d^
4	**1a**	2	0.3	97 ^e^
5	**1b**	2	1	96 ^e^

^a^ Reaction conditions: **1a** (0.1 mmol), Selectfluor, MeCN (0.5 mL). ^b^ room temperature, recovered **1a** (54%). ^c^ reflux, recovered **1a** (35%). ^d^ Ace pressure tube, **1a** (0.4 mmol), MeCN (2 mL). ^e^ Microwave reactor (150 W, ~7 bar, CEM Discover SP).

**Table 2:** Cytotoxicity evaluation using the MTT colorimetric assay.

Compound	IC_50_ MCF-7 (μΜ) ^a^
**1a**	0.810 ± 0.080 ^b^
**2**	0.157 ± 0.001

^a^ IC_50_ represents the compound concentration required for the reduction of the mean cell viability to 50% of the control value after incubation for 72 h at 37 °C. ^b^ IC_50_ values for **1a** were previously obtained under identical conditions [14].

**Table 3:** Formal potentials (*E*^0^′) (±0.010 V) calculated as (*E*_pc_ + *E*_pa_)/2 from cyclic voltammograms recorded at 100 mV·s^−1.^

Compound	*E*^0^′ [V] versus Fc/Fc^+^	
	*E*^0^′ (I)	*E*^0^′ (II)
**1a**	−1.31	−1.72
**2**	−1.21	−1.73

## Supplementary Materials

Supplementary materials are available online. Figures S1–S15: ^1^H, ^13^C and ^19^F-NMR of compounds **2**, **4a**–**4b** and **6a**–**6b**, Figure S16: ^1^H-^13^C HSQC NMR and the optimized geometry of compound **6b**, Figure S17: Viability of the MCF-7 cell line as determined by using the MTT assay for compounds **1a** and **2**.
