# Supplementary material for: Regioselective Fluorination of 7-Oxo-1,2,4-benzotriazines Using Selectfluor
Source: Molecules. 2019 Jan 14;24(2):282. doi: 10.3390/molecules24020282 (PMC6359188; doi:10.3390/molecules24020282)
Supplement: Supplementary file 1 [file molecules-24-00282-s001.pdf]

Supporting Information for

Regioselective Fluorination of 7-Oxo-1,2,4-Benzotriazines using Selectfluor

Styliana I. Mirallai [1], Panayiotis A. Koutentis [2] and Fawaz Aldabbagh [1,3,*]

[1] School of Chemistry, National University of Ireland Galway, University Road, Galway, Ireland; styliana.mirallai@nuigalway.ie (S.I.M.)
[2] Department of Chemistry, University of Cyprus, P.O. Box 20537, 1678 Nicosia, Cyprus; koutenti@ucy.ac.cy (P.A.K.)
[3] Present address: Department of Pharmacy, School of Life Sciences, Pharmacy and Chemistry, Kingston University, Penrhyn Road, Kingston upon Thames, KT1 2EE, UK
* Correspondence: f.aldabbagh@kingston.ac.uk; Tel.: +44-20-8417-2528

Contents

Figures

8-Fluoro-1,3-diphenylbenzo[*e*][1,2,4]triazin-7(1*H*)-one (2)

S1 ^{1}H NMR

S2 ^{13}C NMR

S3 ^{19}F NMR

8-Fluoro-1,3,6-triphenylbenzo[*e*][1,2,4]triazin-7(1*H*)-one (4a)

S4 ^{1}H NMR

S5 ^{13}C NMR

S6 ^{19}F NMR

6-(Benzylthio)-8-fluoro-1,3-diphenylbenzo[e][1,2,4]triazin-7(1*H*)-one (4b)

S7 ^{1}H NMR

S8 ^{13}C NMR

S9 ^{19}F NMR

5-Fluoro-6,8-diphenyl-[1,2,5]thiadiazolo[3',4':5,6]benzo[1,2-*e*][1,2,4]triazin-4(6*H*)-one (6a)

S10 ^{1}H NMR

S11 ^{13}C NMR

S12 ^{19}F NMR

5-Fluoro-6-phenyl-8-(trifluoromethyl)[1,2,5]thiadiazolo[3',4':5,6]-benzo[1,2-*e*][1,2,4]triazin-4(6*H*)-one (6b)

S13 ^{1}H NMR

S14 ^{13}C NMR

S15 ^{19}F NMR

S16 ^{1}H-^{13}C HSQC NMR and geometry optimization

S17 **Viability of MCF-7 cell line as determined using the MTT assay for compounds 1a and 2**

Figure S1. ¹H NMR (500 MHz) of 8-fluoro-1,3-diphenylbenzo[*e*][1,2,4]triazin-7(1*H*)-one (2) in $CDCl_3$.

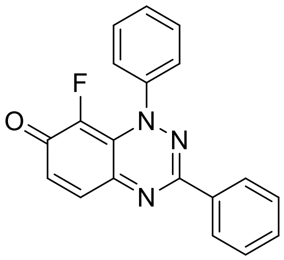

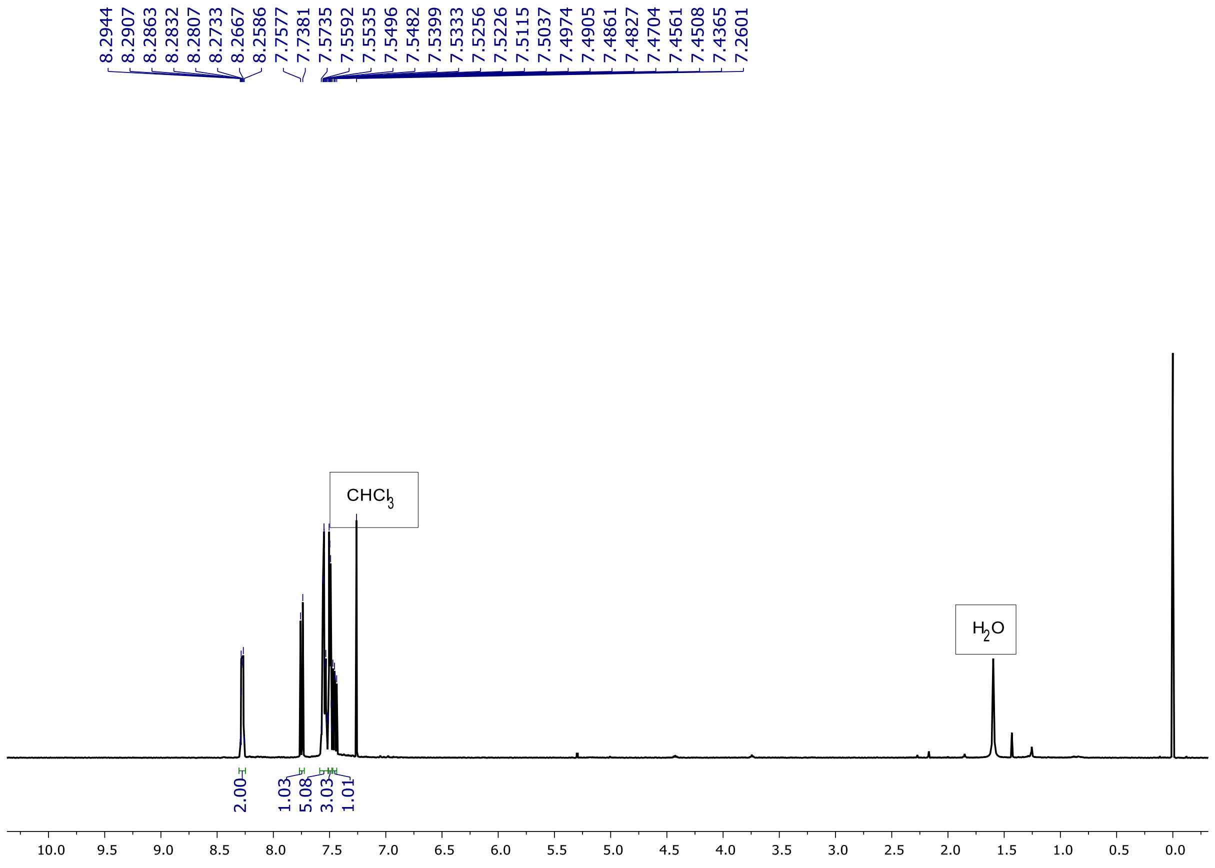

Expansions:

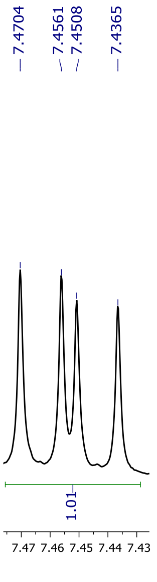

Figure S2. ¹³C NMR (125 MHz) of 8-fluoro-1,3-diphenylbenzo[*e*][1,2,4]triazin-7(1*H*)-one (2) in $CDCl_3$.

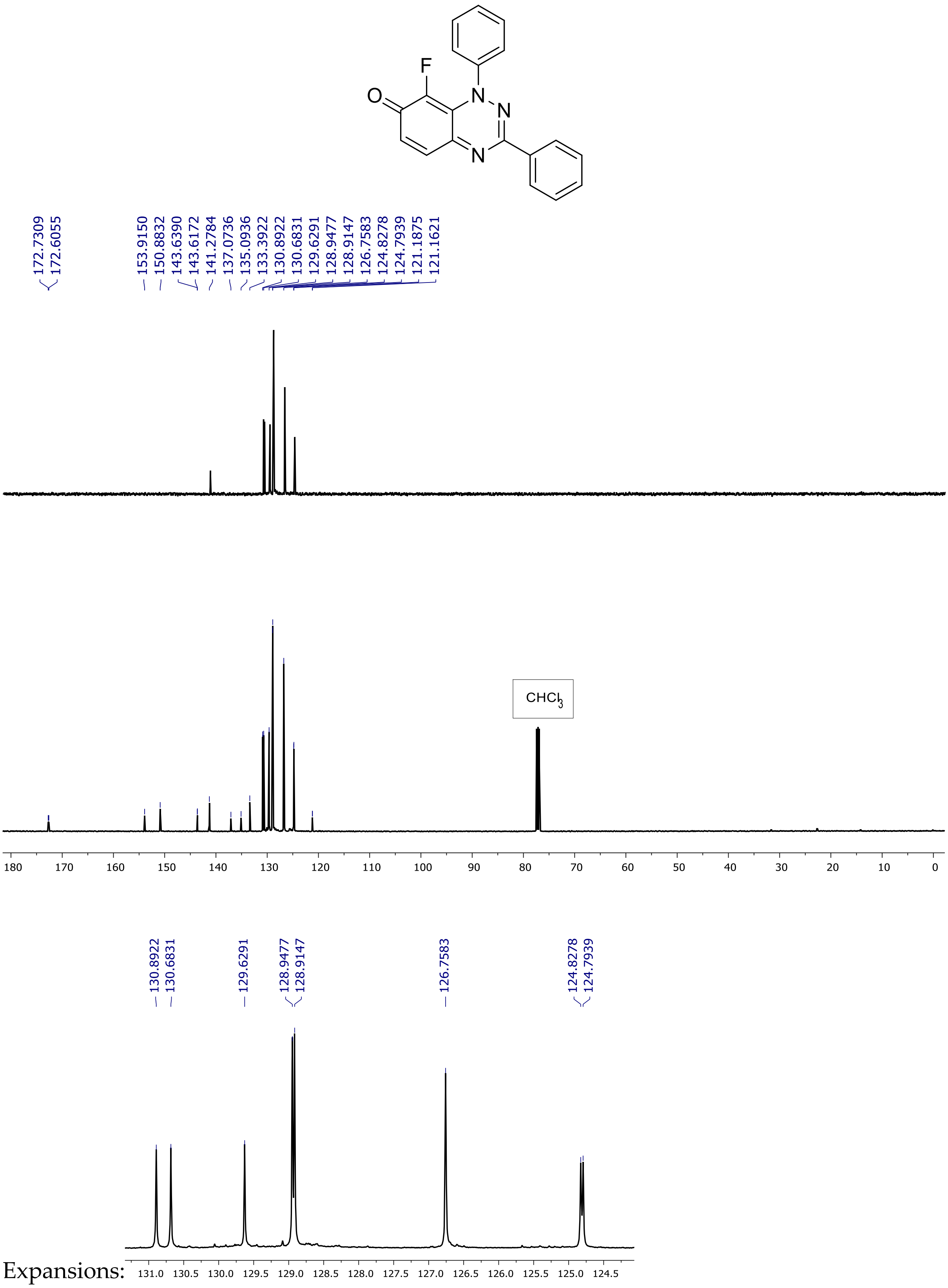

Figure S3. ^{19}F NMR (470 MHz) of 8-fluoro-1,3-diphenylbenzo[*e*][1,2,4]triazin-7(1*H*)-one (2) in $CDCl_3$.

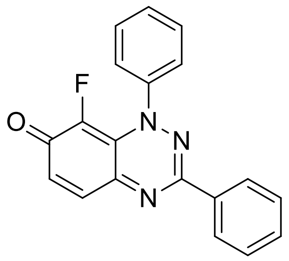

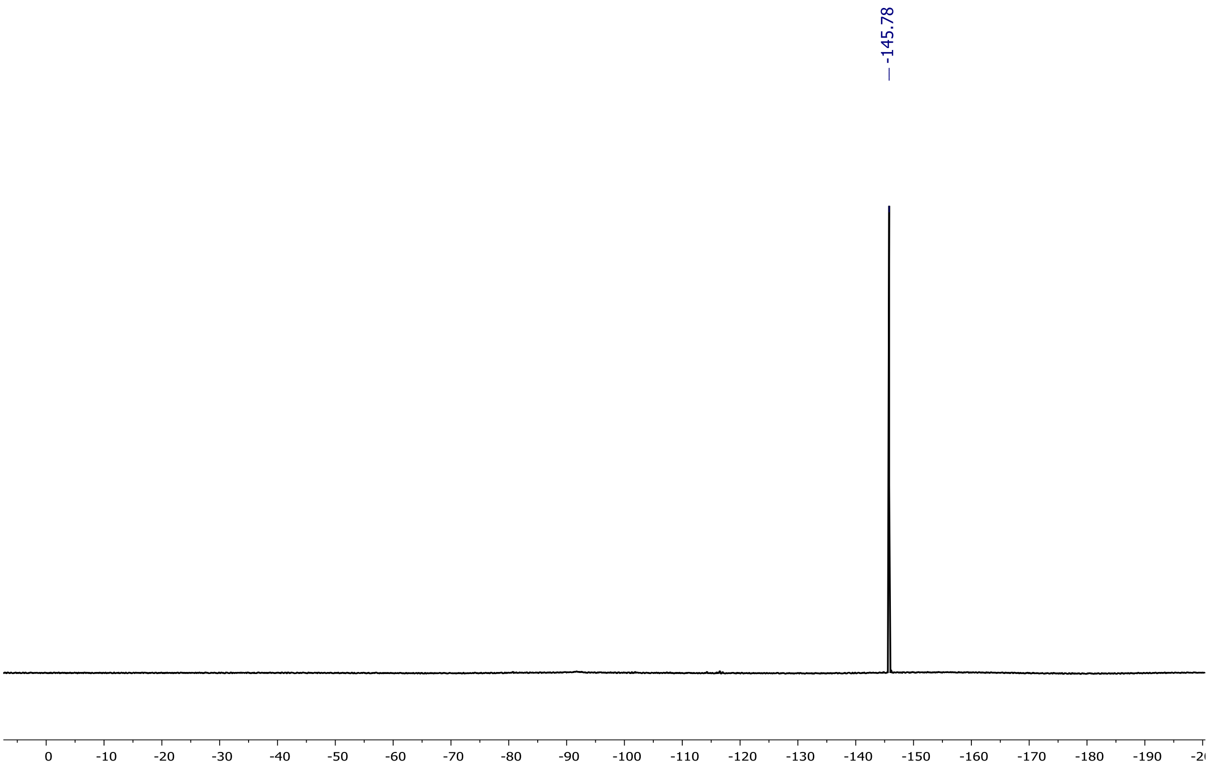

Figure S4. ^{1}**H NMR (500 MHz) of 8-fluoro-1,3,6-triphenylbenzo[*e*][1,2,4]triazin-7(1*H*)-one (4a) in** $CDCl_3$**.**

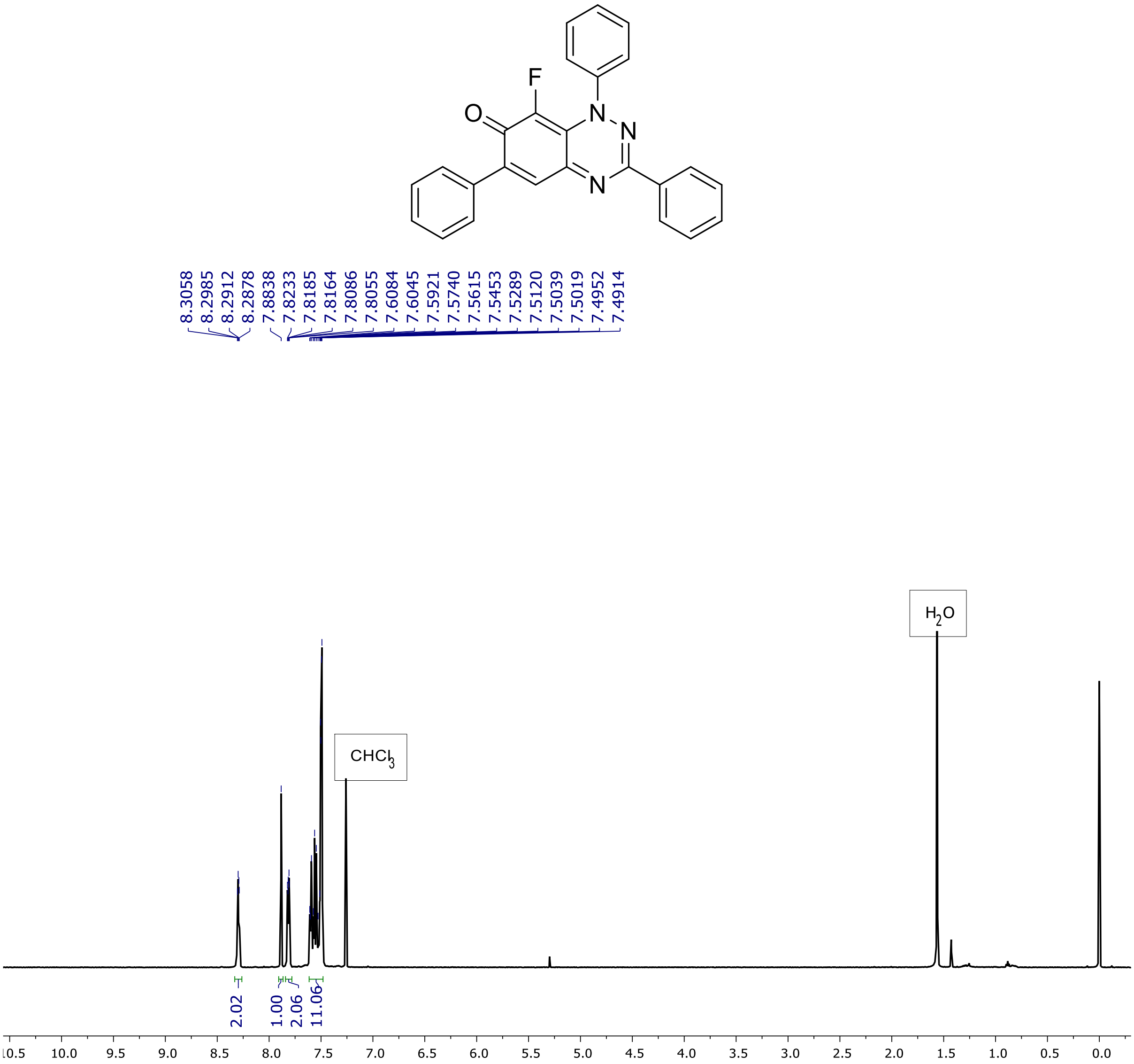

Figure S5. ^{13}**C NMR (125 MHz) of 8-fluoro-1,3,6-triphenylbenzo[*e*][1,2,4]triazin-7(1*H*)-one (4a) in** $CDCl_3$**.**

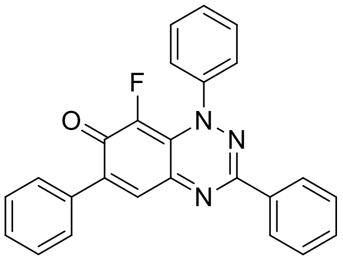

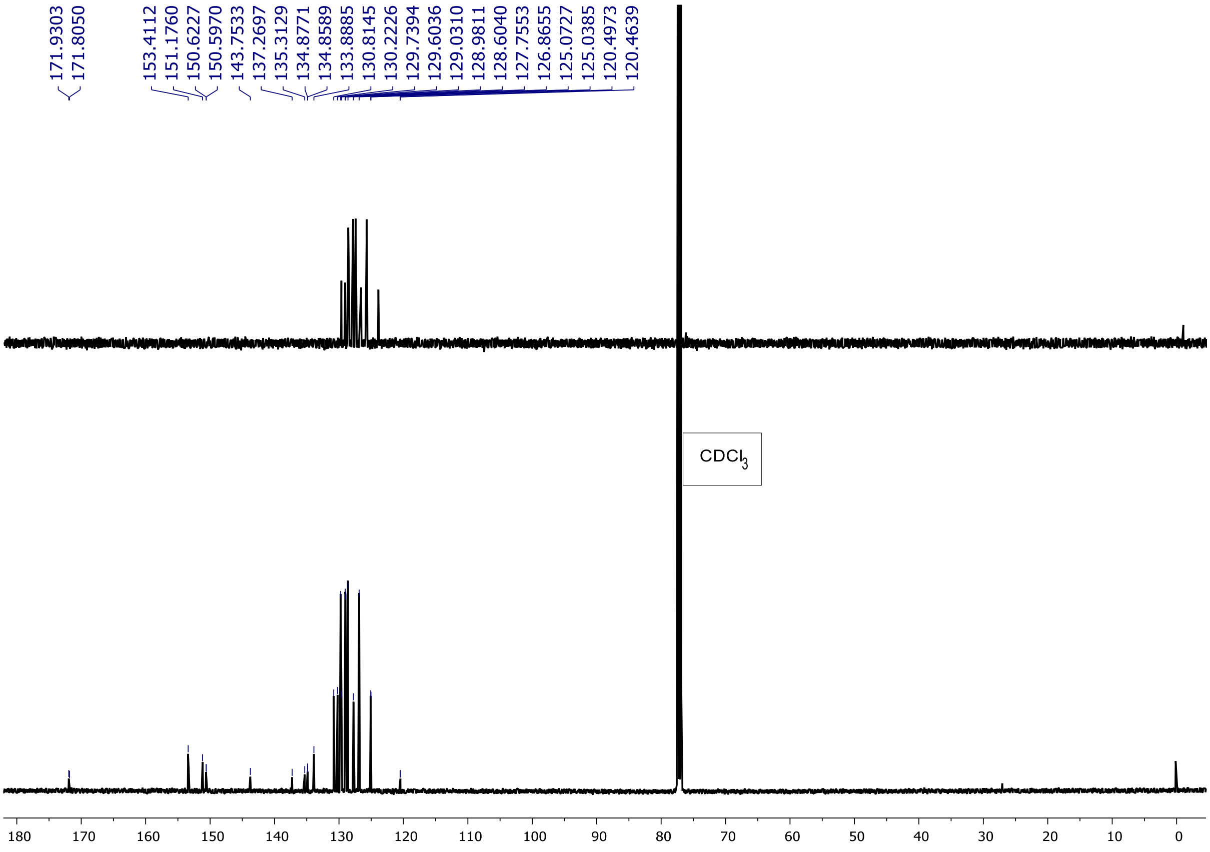

Expansions:

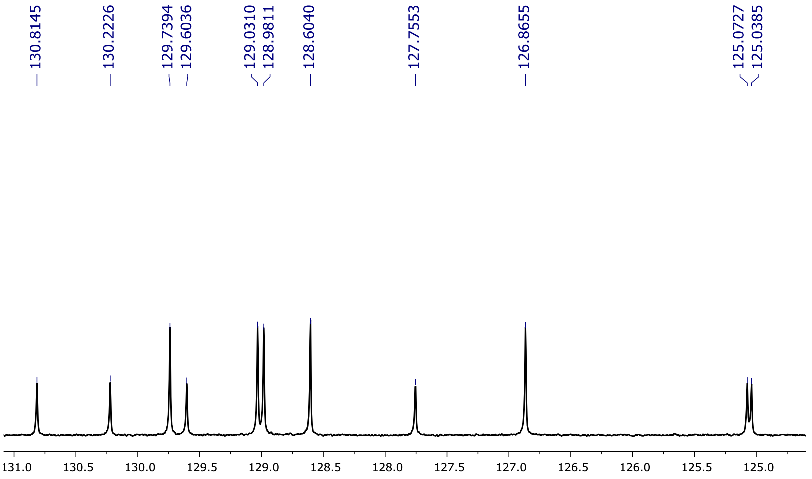

Figure S6. ^{19}F NMR (470 MHz) of 8-fluoro-1,3,6-triphenylbenzo[*e*][1,2,4]triazin-7(1*H*)-one (4a) in $CDCl_3$.

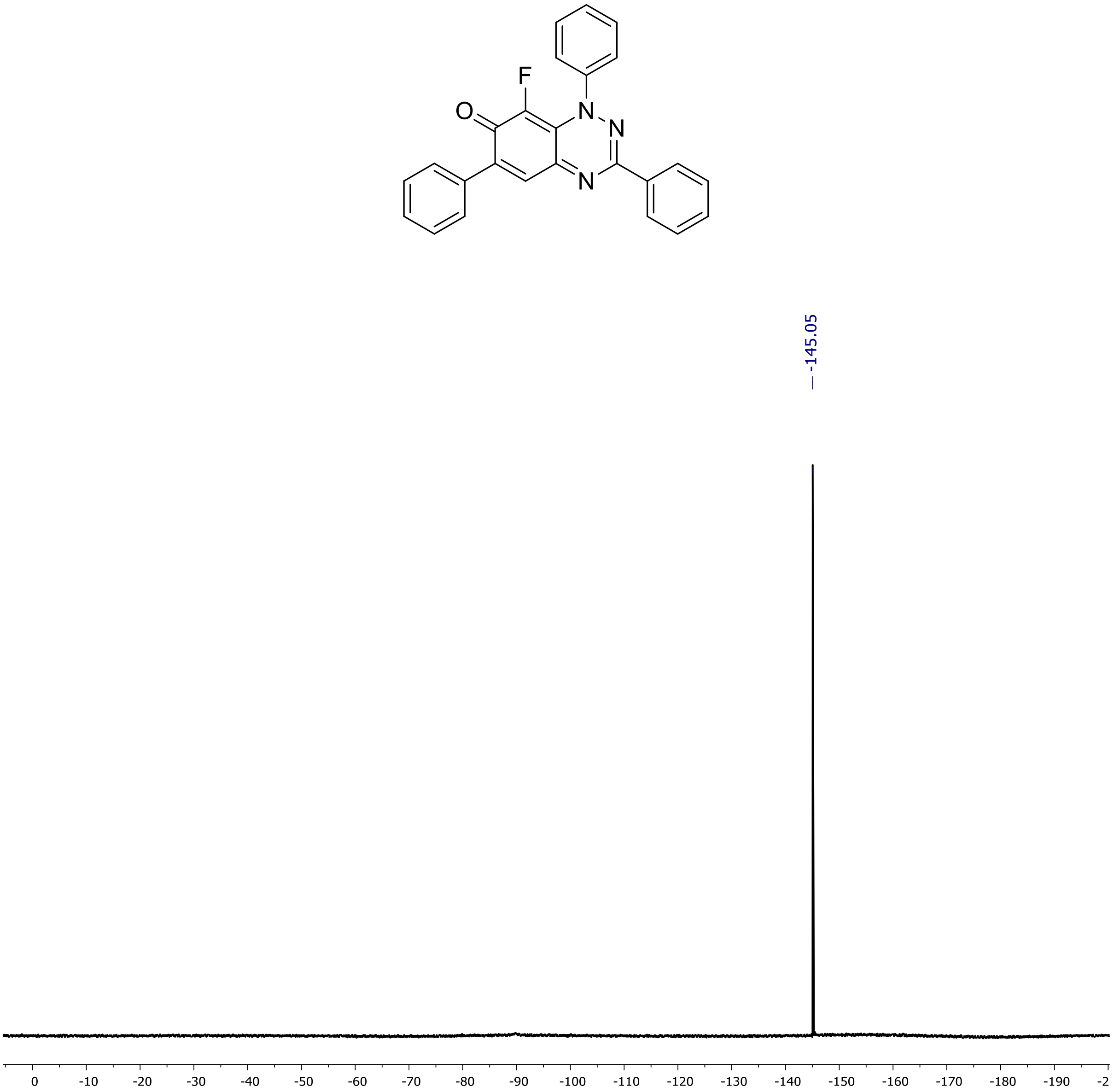

Figure S7. ^{1}H NMR (500 MHz) of 6-(benzylthio)-8-fluoro-1,3-diphenylbenzo[*e*][1,2,4]-triazin-7(1*H*)-one (4b) in $CDCl_3$.

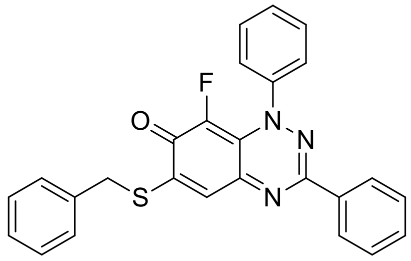

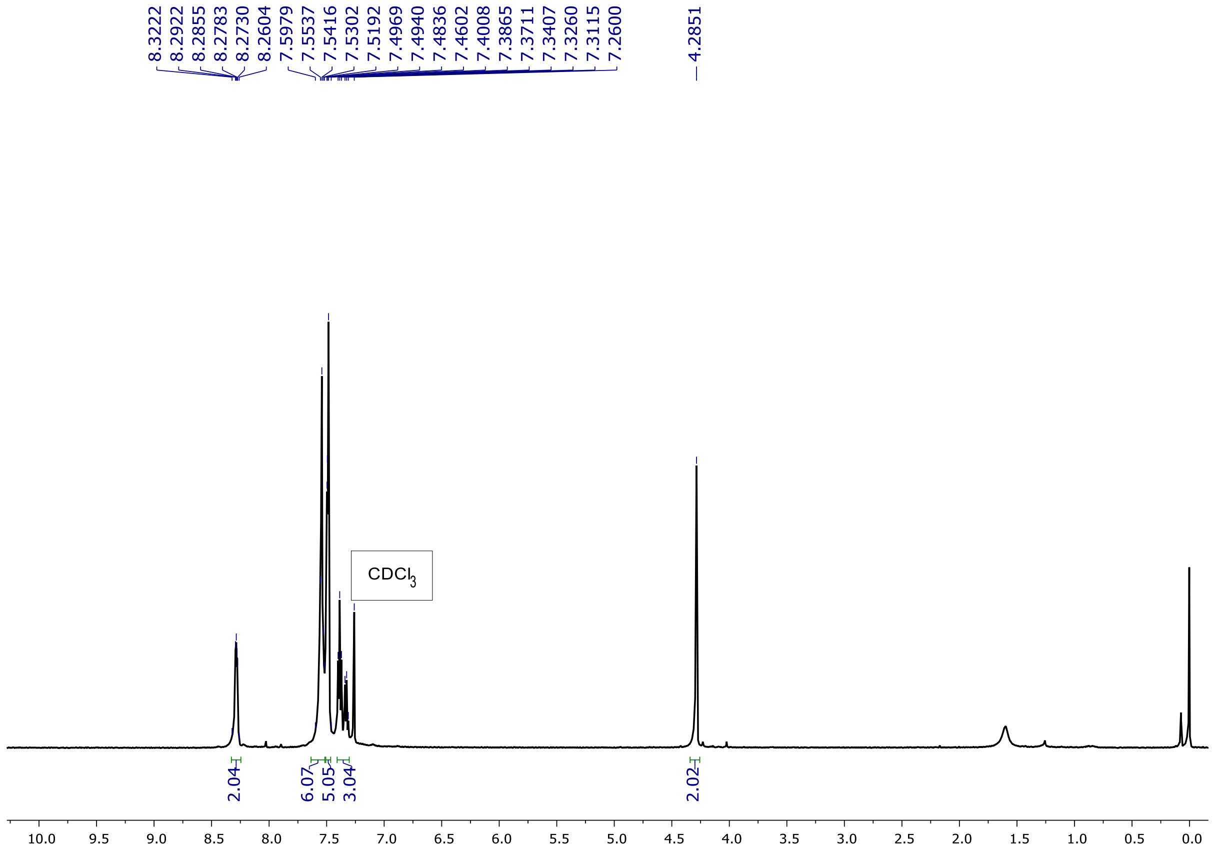

Figure S8. ^{13}C NMR (125 MHz) of 6-(benzylthio)-8-fluoro-1,3-diphenylbenzo[*e*][1,2,4]-triazin-7(1*H*)-one (4b) in $CDCl_3$.

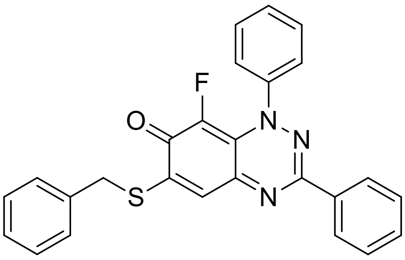

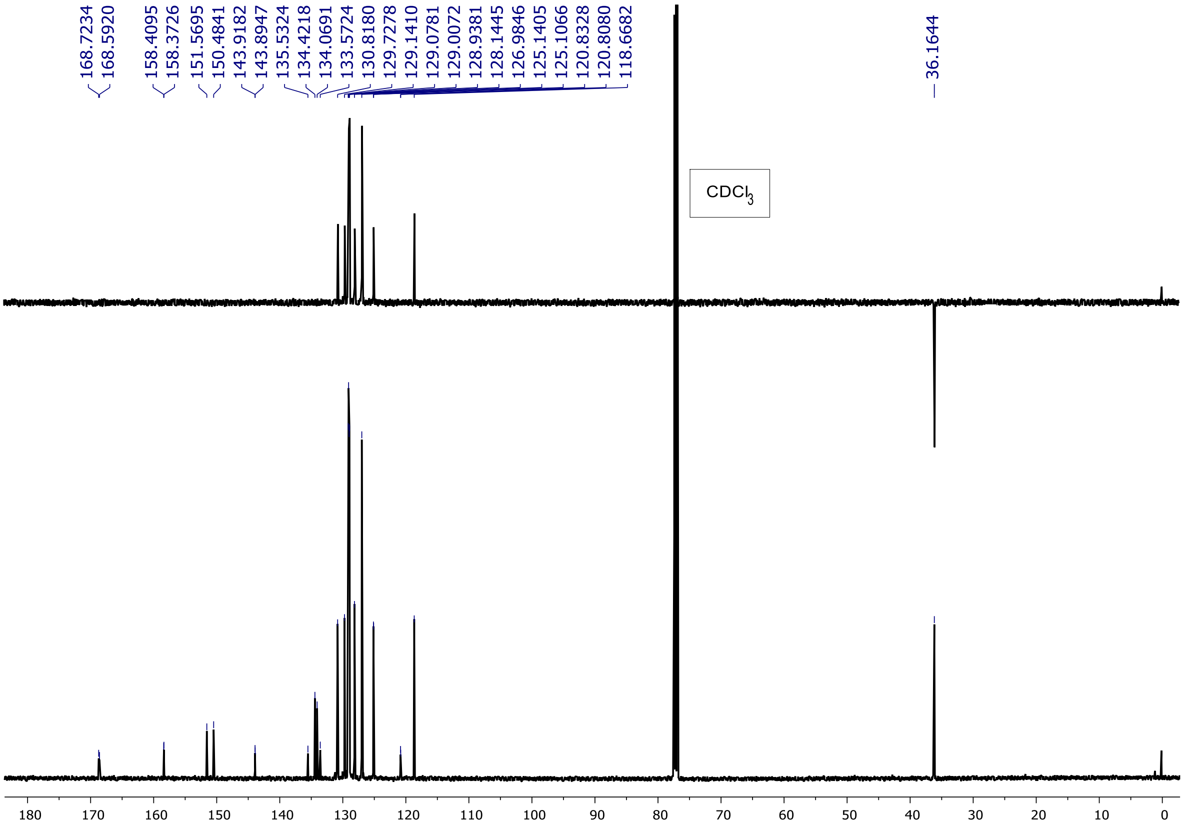

Expansions:

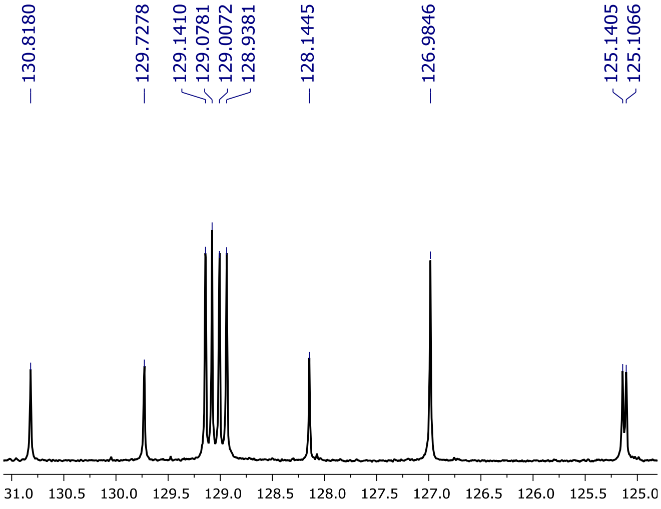

Figure S9. ^{19}F NMR (470 MHz) of 6-(benzylthio)-8-fluoro-1,3-diphenylbenzo[*e*][1,2,4]-triazin-7(1*H*)-one (4b) in $CDCl_3$.

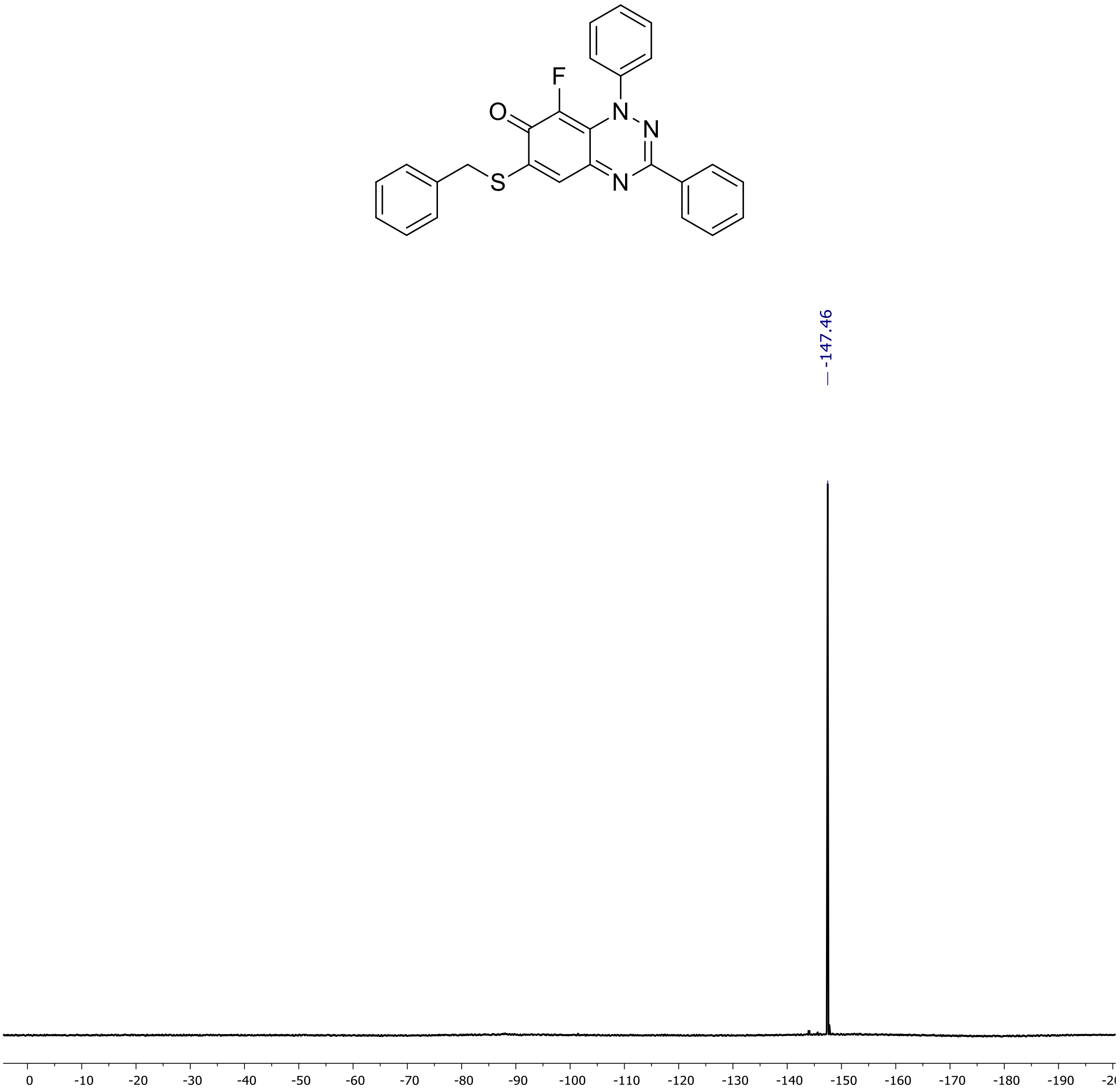

Figure S10. ^{1}H **NMR (500 MHz) of 5-fluoro-6,8-diphenyl[1,2,5]thiadiazolo[3',4':5,6]benzo-[1,2-*e*][1,2,4]triazin-4(6*H*)-one (6a) in CDCl**$_3$**.**

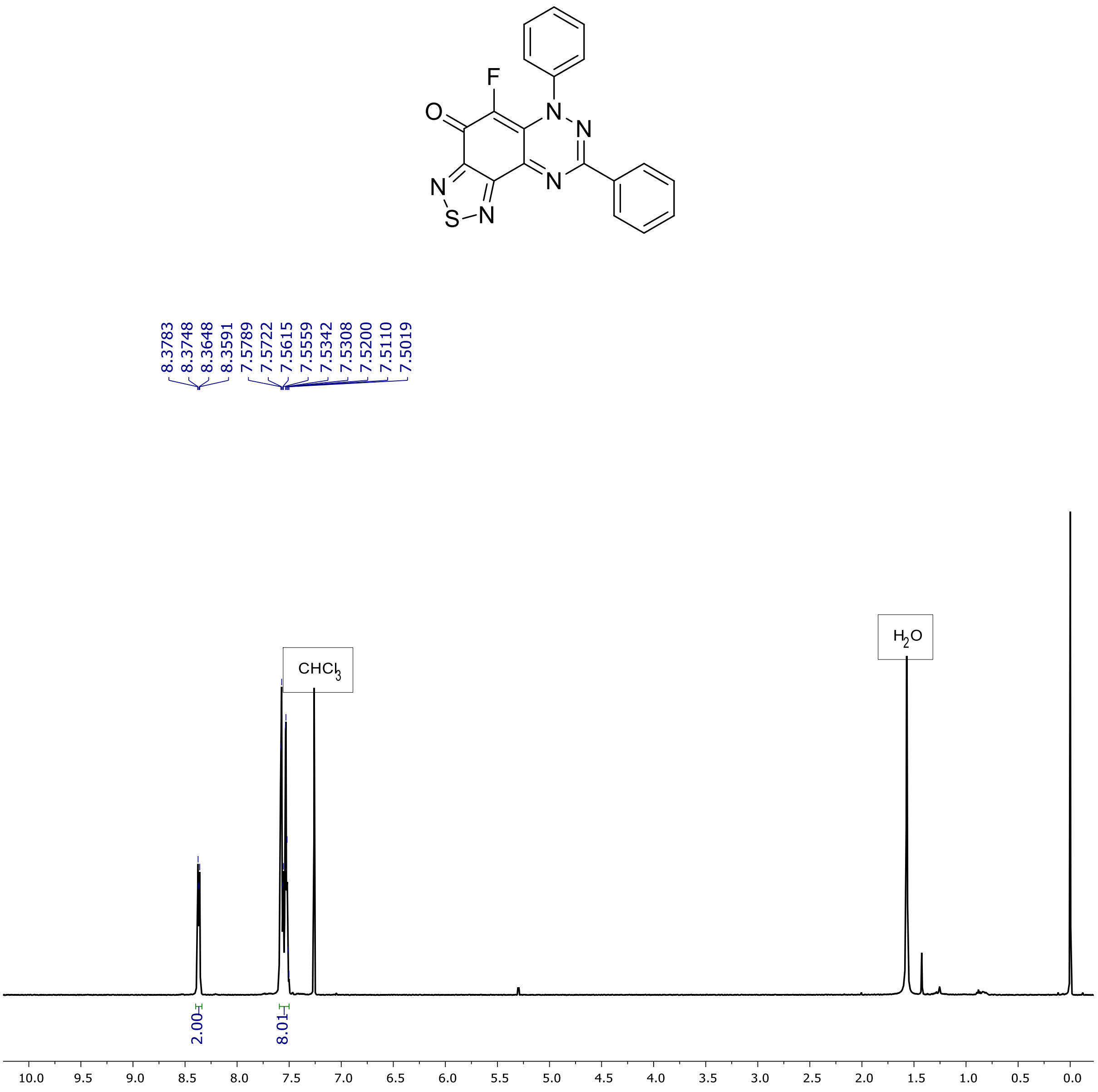

Figure S11. ^{13}C **NMR (125 MHz) of 5-fluoro-6,8-diphenyl[1,2,5]thiadiazolo[3',4':5,6]benzo-[1,2-*e*][1,2,4]triazin-4(6*H*)-one (6a) in** $CDCl_3$**.**

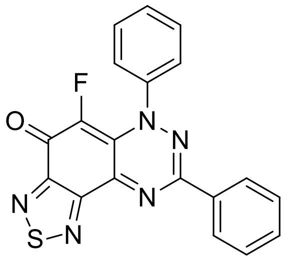

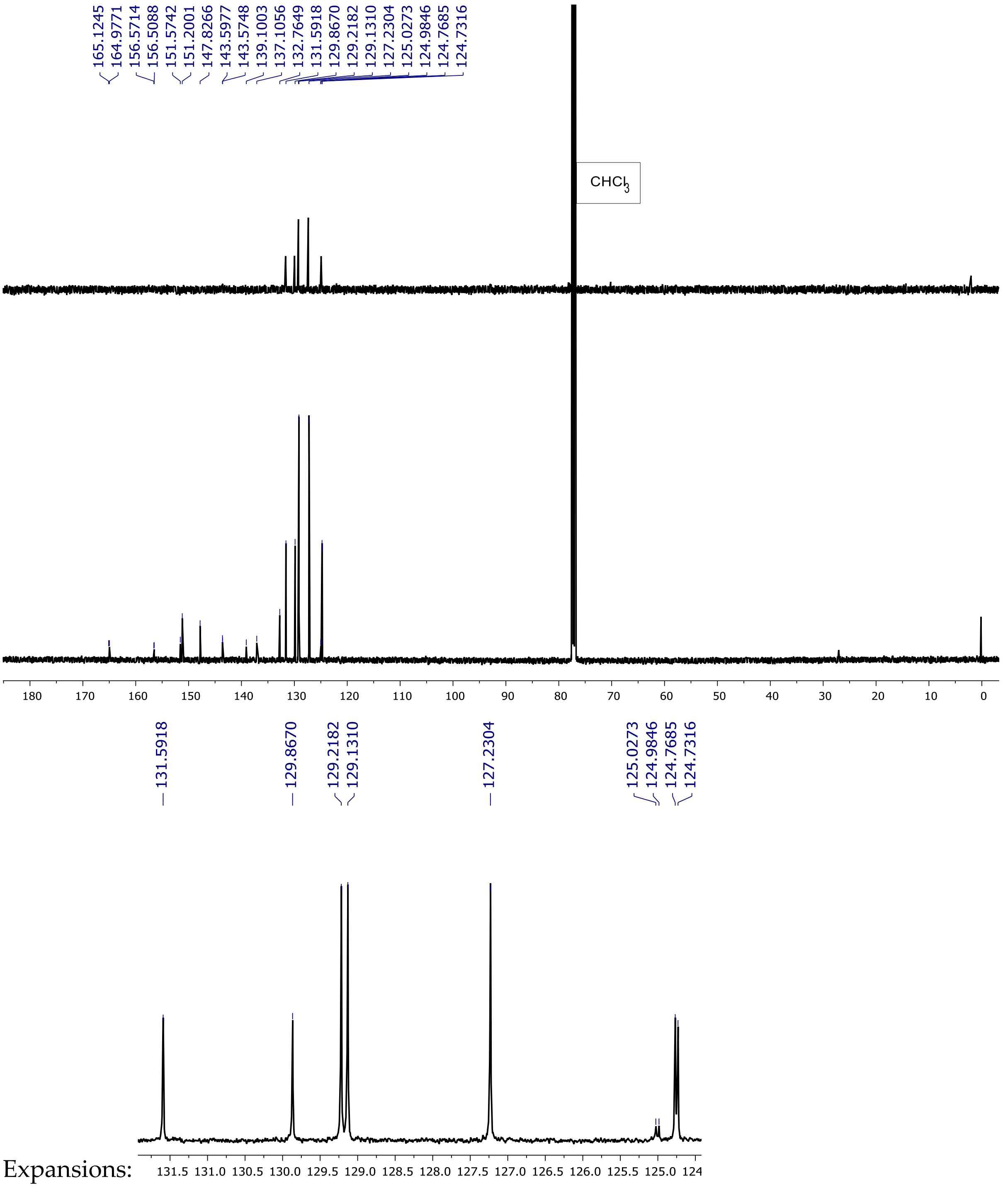

Expansions:

Figure S12. **^{19}F NMR (470 MHz) of 5-fluoro-6,8-diphenyl[1,2,5]thiadiazolo[3',4':5,6]benzo-[1,2-*e*][1,2,4]triazin-4(6*H*)-one (6a) in CDCl$_3$.**

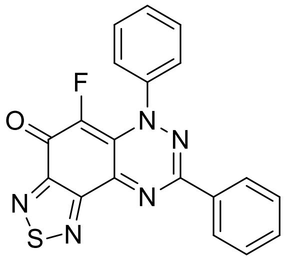

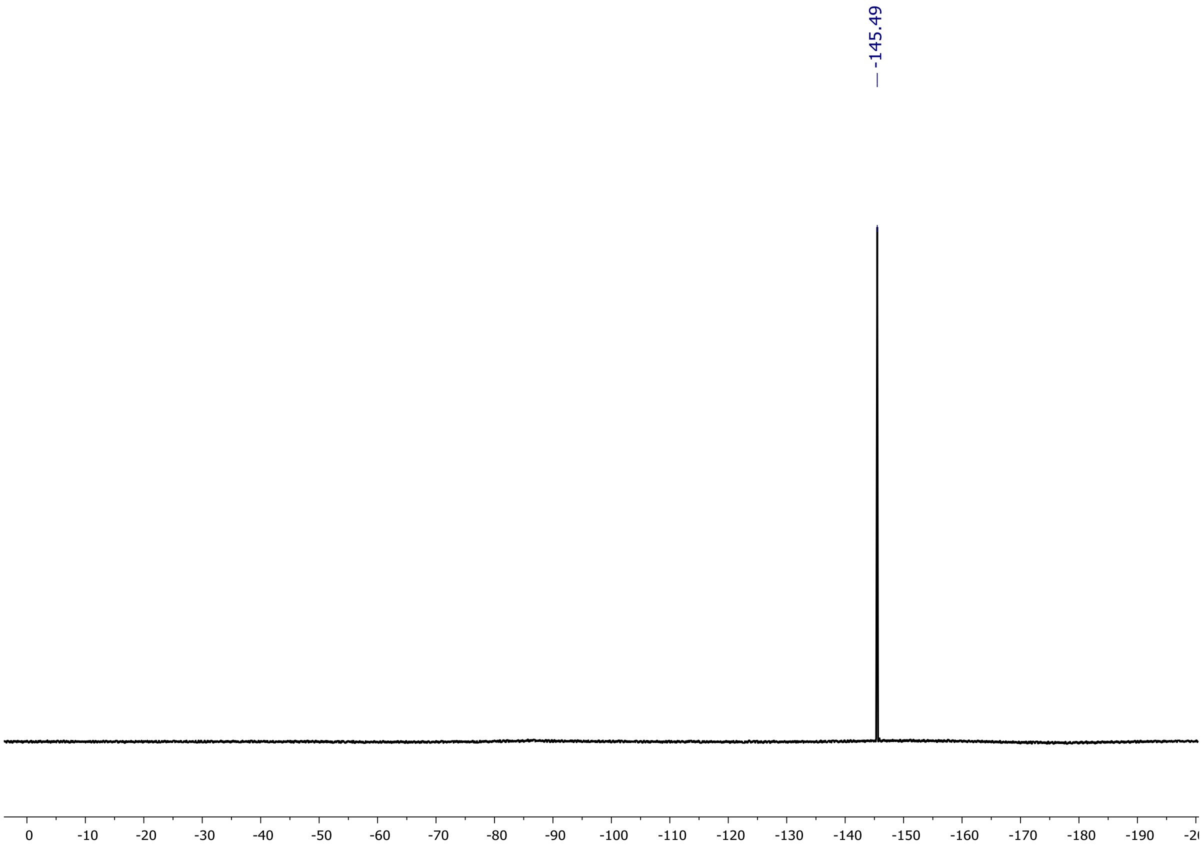

Figure S13. **^{1}H NMR (500 MHz) of 5-fluoro-6-phenyl-8-(trifluoromethyl)[1,2,5]thiadiazolo-[3',4':5,6]benzo[1,2-*e*][1,2,4]triazin-4(6*H*)-one (6b) in $CDCl_3$.**

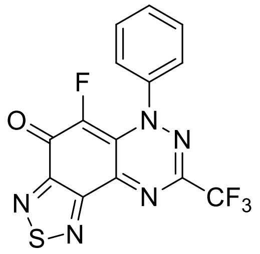

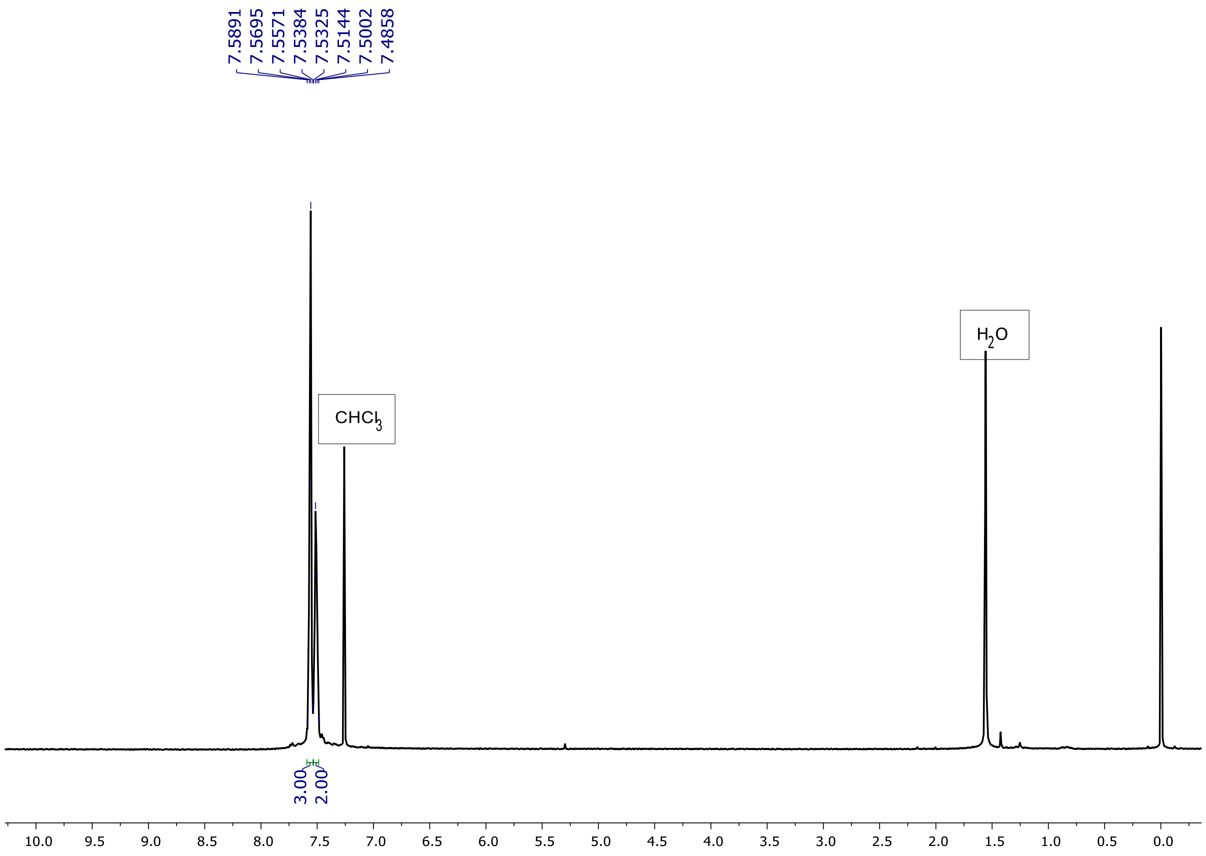

Figure S14. ^{13}C NMR (150 MHz) of 5-fluoro-6-phenyl-8-(trifluoromethyl)[1,2,5]thiadiazolo-[3',4':5,6]benzo[1,2-*e*][1,2,4]triazin-4(6*H*)-one (6b) in $CDCl_3$.

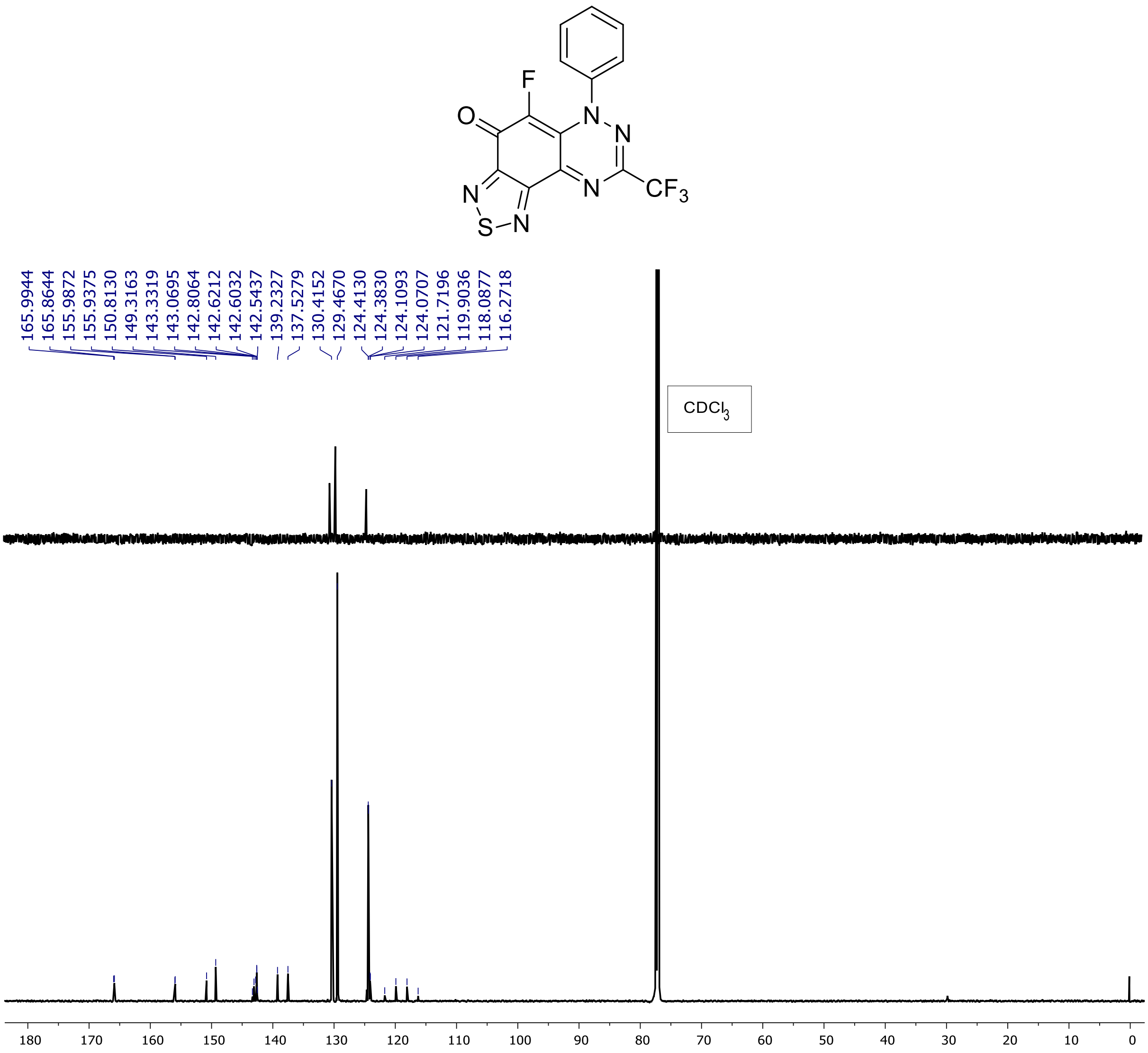

Expansions:

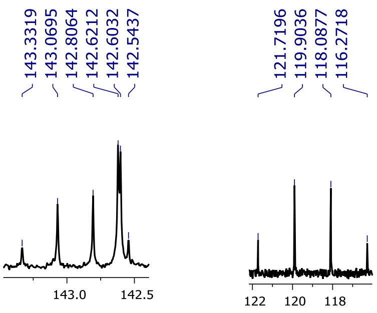

Figure S15. ^{19}F NMR (470 MHz) of 5-fluoro-6-phenyl-8-(trifluoromethyl)[1,2,5]thiadiazolo-[3',4':5,6]benzo[1,2-*e*][1,2,4]triazin-4(6*H*)-one (6b) in $CDCl_3$.

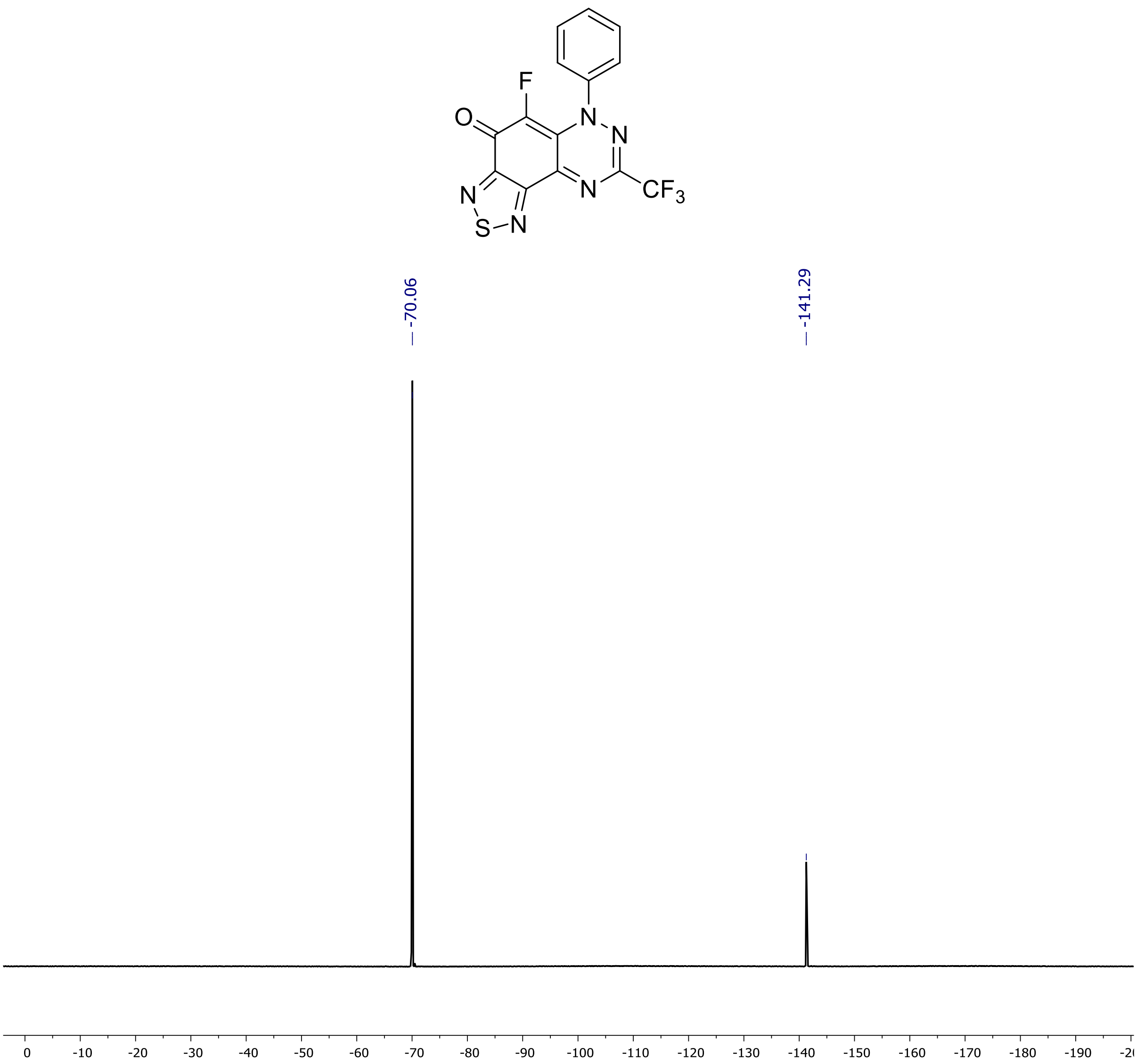

Figure S16. **^{1}H-^{13}C HSQC NMR in $CDCl_3$ and geometry optimization of 5-fluoro-6-phenyl-8-(trifluoromethyl)[1,2,5]thiadiazolo[3',4':5,6]benzo[1,2-*e*][1,2,4]triazin-4(6*H*)-one (6b) performed using Spartan at ground state with Hartree-Fock and 3-21G basis set.**

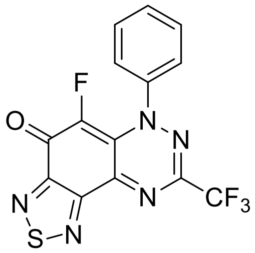

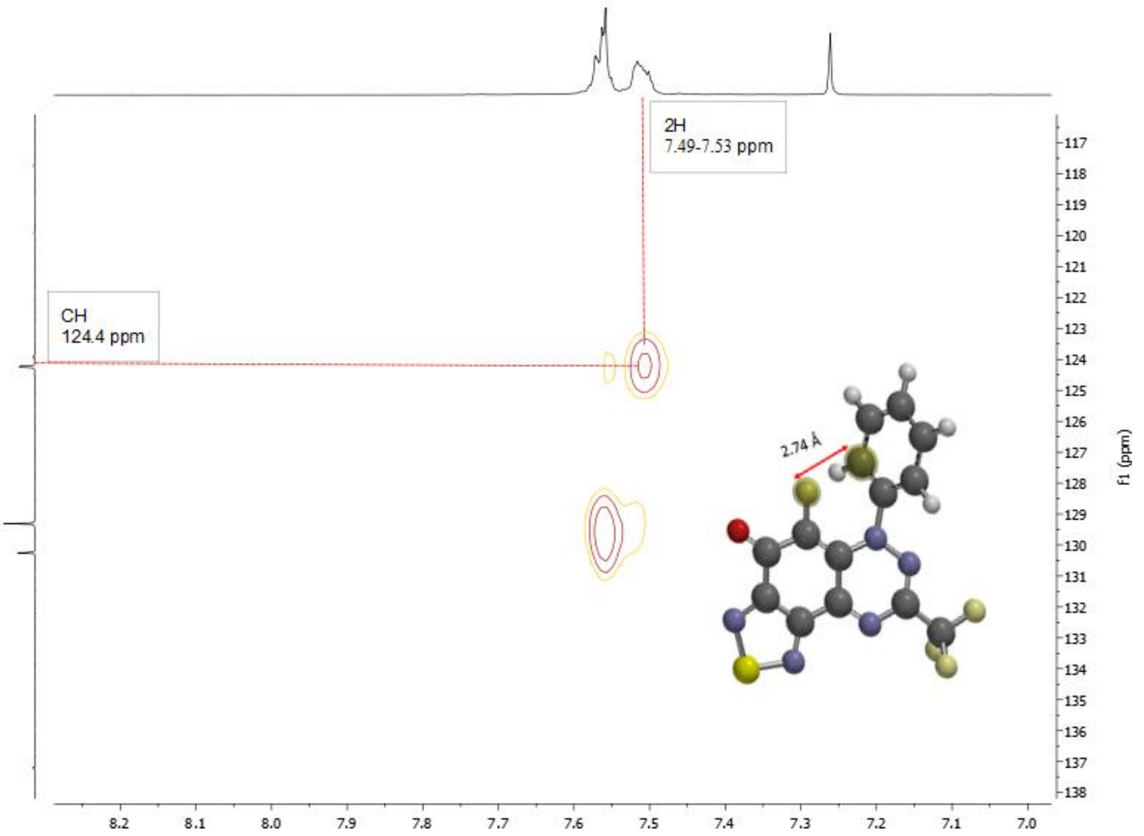

Figure S17. Cell Viability Graph.

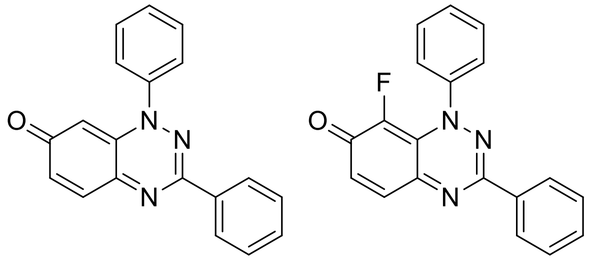

1a **2**

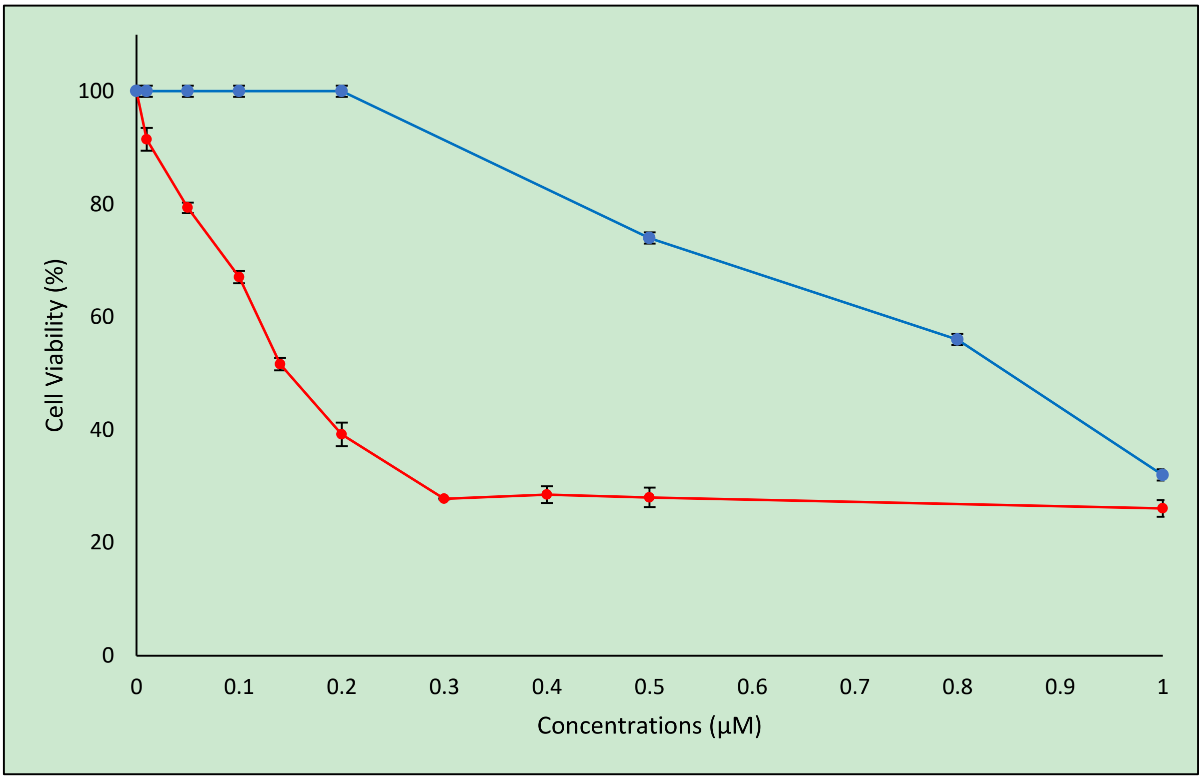

Figure S17. Viability of MCF-7 breast cancer cell line determined using MTT assay following treatment with 1,3-diphenylbenzo[*e*][1,2,4]triazin-7(1*H*)-one (**1a**) (●) and 8-fluoro-1,3-diphenylbenzo[*e*][1,2,4]triazin-7(1*H*)-one (**2**) (●) under aerobic conditions for 72 h at 37 °C. Each data point is the mean of at least three independent experiments. The lines shown are trend lines.
